# Complementary Predictors for Asthma Attack Prediction in Children: Salivary Microbiome, Serum Inflammatory Mediators, and Past Attack History

**DOI:** 10.1111/all.70004

**Published:** 2025-08-18

**Authors:** Shahriyar Shahbazi Khamas, Paul Brinkman, Anne H. Neerincx, Susanne J. H. Vijverberg, Simone Hashimoto, Jelle M. Blankestijn, Jan Willem Duitman, Tamara Dekker, Barbara S. Smids, Suzanne W. J. Terheggen‐Lagro, René Lutter, Nariman K. A. Metwally, Fleur Sondaal, Eric G. Haarman, Peter J. Sterk, Ian M. Adcock, Charles Auffray, Corinna Bang, Aruna T. Bansal, Heike Buntrock‐Döpke, Klaus Bønnelykke, Andrew Bush, Bo Lund Chawes, Kian Fan Chung, Paula Corcuera‐Elosegui, Sven‐Erik Dahlén, Ratko Djukanovic, Louise J. Fleming, Stephen J. Fowler, Andre Franke, Urs Frey, Mario Gorenjak, Susanne Brandstetter, Susanne Harner, Gunilla Hedlin, Michael Kabesch, Nazanin Zounemat‐Kermani, Parastoo Kheirolldein, Alexander Kiefer, Jon R. Konradsen, Aletta D. Kraneveld, Leyre López‐Fernández, Clare S. Murray, Björn Nordlund, Maria Pino‐Yanes, Uroš Potočnik, Graham Roberts, Jakob Stokholm, Søren Johannes Sørensen, Olaia Sardón‐Prado, Dominick E. Shaw, Florian Singer, Ana R. Sousa, Jonathan Thorsen, Antoaneta A. Toncheva, Nadja H. Vissing, Christine Wolff, Mahmoud I. Abdel‐Aziz, Anke H. Maitland‐van der Zee, the SysPharmPediA and U‐BIOPRED Consortia

**Affiliations:** ^1^ Amsterdam UMC Location University of Amsterdam Department of Pulmonary Medicine Amsterdam the Netherlands; ^2^ Amsterdam Institute for Infection and Immunity Amsterdam the Netherlands; ^3^ Amsterdam Public Health Amsterdam the Netherlands; ^4^ Department of Paediatric Pulmonary Medicine, Emma Children's Hospital, Amsterdam UMC University of Amsterdam Amsterdam the Netherlands; ^5^ Department of Experimental Immunology Amsterdam UMC Location University of Amsterdam Amsterdam the Netherlands; ^6^ National Heart and Lung Institute Imperial College London London UK; ^7^ Royal Brompton & Harefield Hospital London UK; ^8^ European Institute for Systems Biology and Medicine, CIRI UMR5308, CNRS‐ENS‐UCBL‐INSERM Lyon France; ^9^ Institute of Clinical Molecular Biology Christian‐Albrechts‐University of Kiel Kiel Germany; ^10^ Acclarogen Ltd. St. John's Innovation Centre Cambridge UK; ^11^ Science and Development Campus Regensburg (WECARE), university Children's Hospital Regensburg (KUNO) at the Hospital St. Hedwig of the Order of St. John University of Regensburg Regensburg Germany; ^12^ COPSAC, Copenhagen Prospective Studies on Asthma in Childhood Copenhagen University Hospital—Herlev and Gentofte Copenhagen Denmark; ^13^ Division of Pediatric Respiratory Medicine Hospital Universitario Donostia San Sebastián Spain; ^14^ Clinical Lung and Allergy Research Unit, Department of Medicine Huddinge Karolinska Institutet Stockholm Sweden; ^15^ Integrative Metabolomics Unit, Institute of Environmental Medicine Karolinska Institutet Stockholm Sweden; ^16^ Department of Respiratory Medicine and Allergology Karolinska University Hospital Stockholm Sweden; ^17^ Southampton Biomedical Research Centre, University Hospital Southampton NHS Foundation Trust and Clinical and Experimental Sciences and Human Development and Health University of Southampton Southampton UK; ^18^ Division of Infection, Immunity and Respiratory Medicine, School of Biological Sciences, Faculty of Biology, Medicine and Health University of Manchester Manchester UK; ^19^ Manchester Academic Health Science Centre and National Institute for Health and Care Research Biomedical Research Centre Manchester University Hospitals NHS Foundation Trust Manchester UK; ^20^ University Children's Hospital Basel UKBB University of Basel Basel Switzerland; ^21^ Center for Human Molecular Genetics and Pharmacogenomics, Faculty of Medicine University of Maribor Maribor Slovenia; ^22^ Department of Pediatric Pneumology and Allergy University Children's Hospital Regensburg (KUNO) at the Hospital St.Hedwig of the Order of St. John, University of Regensburg Regensburg Germany; ^23^ Astrid Lindgren Children's Hospital Karolinska University Hospital Stockholm Sweden; ^24^ Department of Women's and Children's Health Karolinska Institute Stockholm Sweden; ^25^ Division of Pharmacology, Utrecht Institute for Pharmaceutical Sciences, Faculty of Science Utrecht University Utrecht the Netherlands; ^26^ Genomics and Health Group, Department of Biochemistry, Microbiology, Cell Biology and Genetics Universidad de La Laguna La Laguna Santa Cruz de Tenerife Spain; ^27^ CIBER de Enfermedades Respiratorias Instituto de Salud Carlos III Madrid Spain; ^28^ Instituto de Tecnologías Biomédicas (ITB) Universidad de La Laguna (ULL) La Laguna Spain; ^29^ Laboratory for Biochemistry, Molecular Biology and Genomics, Faculty of Chemistry and Chemical Engineering University of Maribor Maribor Slovenia; ^30^ Department for Science and Research University Medical Centre Maribor Maribor Slovenia; ^31^ Department of Food Science University of Copenhagen Frederiksberg Denmark; ^32^ Section of Microbiology, Department of Biology University of Copenhagen Copenhagen Denmark; ^33^ Department of Pediatrics University of the Basque Country (UPV/EHU) San Sebastián Spain; ^34^ Leicester NIHR Biomedical Research Centre and Department of Respiratory Sciences University of Leicester Leicester UK; ^35^ Division of Paediatric Pulmonology and Allergology, Department of Paediatrics and Adolescent Medicine Medical University of Graz Graz Austria; ^36^ Division of Paediatric Respiratory Medicine and Allergology, Department of Paediatrics, Inselspital Bern University Hospital, University of Bern Bern Switzerland; ^37^ Respiratory Therapeutic Unit GlaxoSmithKline London UK; ^38^ Dept of Clinical Medicine University of Copenhagen Copenhagen Denmark

**Keywords:** 16S rRNA, asthma, biomarker, exacerbations, precision medicine, saliva

## Abstract

**Background:**

Early identification of children at risk of asthma attacks is important for optimizing treatment strategies. We aimed to integrate salivary microbiome and serum inflammatory mediator profiles with asthma attacks history to develop a comprehensive predictive model for future attacks.

**Methods:**

This study contained a discovery (SysPharmPediA) and a replication phase (U‐BIOPRED). School‐aged children with asthma were classified into at risk and no‐risk groups, based on the presence or absence of one or more severe attacks during one‐year follow‐up. Prediction models were developed using random forest on the training set (70%) with data on past asthma attacks, microbiome composition, serum inflammatory mediator levels, and their combinations and then tested on the rest of the population (30%). Outcomes were replicated in a subset of children with severe asthma from U‐BIOPRED.

**Results:**

Complete data were available for 154 children (SysPharmPediA = 121, U‐BIOPRED = 33). In discovery, the model based on past attacks resulted in an area under the receiving characteristic curve (AUROCC) ~ 0.7. Models including six salivary bacteria or six inflammatory mediators achieved similar results. The combined model incorporating seven features, past asthma attacks, *Capnocytophaga*, *Corynebacterium*, and *Cardiobacterium*, TIMP‐4, VEGF, and MIP‐3β achieved the highest accuracy with AUROCC ~0.87. The combined model in the U‐BIOPRED limited to available inflammatory mediators (VEGF), and incorporating past asthma attacks, *Capnocytophaga, Corynebacterium,* and *Cardiobacterium*, resulted in an AUROCC of 0.84.

**Conclusion:**

Serum inflammatory mediators and salivary microbiome complement asthma attacks history for predicting future attacks. These results highlight the imperative for continued investigation into oral microbiota and its interaction with the immune system.

## Graphical abstract

In this two‐phase study, school‐aged children were classified as at risk or no‐risk based on severe attacks during one‐year follow‐up. Four types of prediction models were developed. Salivary bacteria and serum inflammatory markers, while informative alone, complement past asthma attacks in predicting future attack risk. Combined model outperformed single‐data models, achieving AUROCC ~0.87 in discovery and ~0.84 in replication. AUC, area under the curve; MIP‐3β, monocyte inflammatory protein 3β; SysPharmPediA, systems pharmacology approach to uncontrolled pediatric asthma; TIMP‐4, tissue inhibitor of metalloproteinase 4; U‐BIOPRED, unbiased biomarkers for the prediction of respiratory disease outcomes; VEGF, vascular endothelial growth factor.

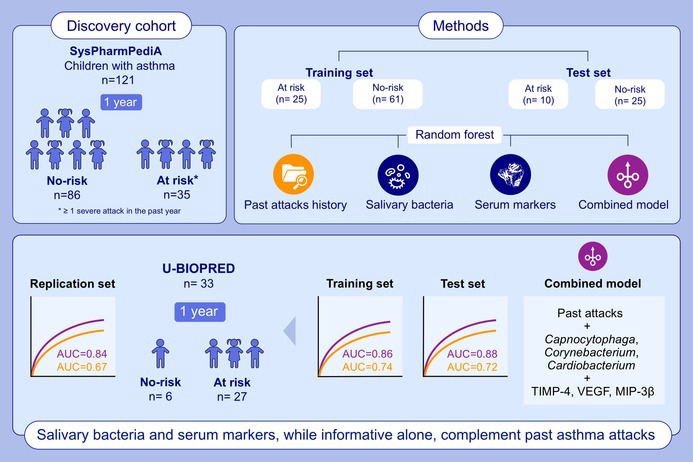

**Abbreviations**
AUROCCs
: area under the receiver operating characteristic curves
FE_NO_

: fractional exhaled nitric oxide
H₂S: hydrogen sulphidesulfide
LPS
: lipopolysaccharides
LTA
: lipoteichoic acids
MIP)‐3β: monocyte inflammatory protein
MMP)‐9: TIMP‐1, and matrix metalloproteinase
RAGE
: receptor for advanced glycation end products
RF
: random forest
SCFAs
: short‐chain fatty acids
SysPharmPediA
: Systems Pharmacology Approach to Uncontrolled PediatricPaediatric Asthma
TIMP)‐4: tissue inhibitor of metalloproteinase
U‐BIOPRED
: Unbiased Biomarkers for the Prediction of Respiratory Disease Outcomes
VEGF
: vascular endothelial growth factor

## 1 Introduction

Asthma is the most common chronic disease in children, with asthma attacks (exacerbations) posing significant risks to their health, as well as imposing a considerable burden on families and the healthcare system [1]. The underlying mechanisms driving asthma in children remain incompletely understood, stemming from a complex interplay of various factors, such as genetic predisposition, environmental exposures, and the microbiota [2, 3]. Looking beyond the immediate symptoms, these attacks may contribute to a decline in lung function over time [4]. Therefore, the development of novel strategies to prevent asthma attacks in children is paramount.

Preventing severe asthma attacks hinges on early identification of children at high risk, enabling the development of tailored treatment plans that can potentially prevent such attacks. While a recent severe attack is one of the most reliable predictors of future episodes [5], this predictor is limited as an early warning system, as it fails to capture the underlying pathophysiological changes that precede an attack. Moreover, relying solely on past asthma attacks does not help prevent the initial attack and is further constrained by seasonal variations and lack of precision, while also overlooking other contributing factors [6, 7]. Biomarkers offer a promising alternative, as they can reflect these biological processes, providing earlier detection of high‐risk individuals and facilitating more precise, targeted interventions that address the root causes of asthma attacks [5].

Recent years have seen a surge in interest regarding the oral (salivary) microbiome as a potential player in asthma pathogenesis and management. Traditionally, research has focused on the lower airway and gut microbiomes, but accumulating evidence highlights the oral cavity as a dynamic microbial reservoir that both reflects and influences respiratory health [8]. The oral cavity forms a crucial interface between the external environment and the internal mucosal surfaces of both the respiratory and gastrointestinal tracts. The oral microbiota, the body's second most abundant bacterial flora after the intestinal tract, is not only easily accessible for sampling but also demonstrates distinct compositional shifts in asthma, making it a promising non‐invasive biomarker for immunomodulation, asthma risk, treatment response, and severity [9, 10, 11, 12, 13].

The oral cavity acts as a source of microbes that are frequently aspirated into the lungs, where they can alter the lung microenvironment [14, 15]. This microbial transfer plays a crucial role in shaping local immune responses, potentially influencing the progression and severity of asthma [16]. Furthermore, oral microbes can influence the gut microbiome via swallowing, with some bacteria surviving gastric passage to affect gut microbial communities [17, 18, 19]. Oral dysbiosis has been linked to gut and systemic inflammation, both of which are relevant to asthma pathogenesis [8].

The concept of the oral–gut–lung axis describes a multidirectional network of microbial and immune interactions in which oral microbes may impact both gut and lung health directly through microbial translocation and indirectly via immune modulation [8, 20, 21, 22]. This tri‐directional relationship underscores the oral microbiome's potential role not only as a reflection of airway and gut health but also as a modulator of immune responses relevant to asthma pathophysiology [8, 12, 20, 23, 24].

Given the susceptibility of the microbiome to modulation through changes in lifestyle, diet, and medications (including antibiotics, prebiotics and/or probiotics), it holds promise as a potential therapeutic target for individuals with asthma [25]. Considering the intricate relationship between the microbiome and the immune system [26, 27, 28], examining how oral bacterial communities interact with the host's immune responses is essential when predicting asthma clinical outcomes.

The immune system plays a crucial role in asthma pathophysiology, and interactions between specific bacteria and immune mediators can significantly influence inflammation and asthma attacks [28, 29]. While serum inflammatory mediators provide valuable insights into immune system activity, may help phenotype patients, guide treatment decisions, and support understanding of asthma pathophysiology, these markers often reflect systemic inflammation rather than localized airway processes and may not fully capture the complexity of asthma attacks [30, 31]. Understanding the microbial and immune interactions can provide deeper insight into the mechanisms driving asthma attacks, enabling better prediction of disease progression and response to treatment by incorporating baseline microbiome data and systemic immune responses [13, 32]. This holistic approach can lead to more robust predictive models, significantly enhancing the accuracy of clinical predictions beyond what is possible by studying previous asthma attacks alone.

Recently, we analyzed saliva samples from the SysPharmPediA (Systems Pharmacology Approach to Uncontrolled Paediatric Asthma) cohort using 16S ribosomal RNA gene sequencing to elucidate the role of the salivary bacteria in school‐age children with asthma. We found that a set of bacterial genera differentiates between controlled and uncontrolled asthma with an average classification accuracy of 81%, where asthma control was defined based on past exacerbations and the asthma control test [9]. Similarly, in another study involving the U‐BIOPRED (Unbiased Biomarkers for the Prediction of Respiratory Disease Outcomes) pediatric cohort, we demonstrated that oropharyngeal bacteria profiles were associated with distinct clinical characteristics in children, including results of spirometry, atopic dermatitis, allergic sensitization, and the risk of future asthma attacks [33]. Building upon these findings, we hypothesized that salivary bacterial diversity and composition are associated with the risk of future asthma attacks in children. In this study, we aimed to integrate saliva microbiome and serum inflammatory mediator profiles with asthma attacks history to develop a comprehensive predictive model. This approach aligns with current recommendations that predictive models of severe asthma attacks should include a combination of epidemiological/clinical data, as well as objective biomarkers [5].

## 2 Methods

### 2.1 Study Design

This prospective observational international multicenter study comprised two phases: discovery (SysPharmPediA) and replication (pediatric U‐BIOPRED cohort). All participants had to be between 6 and 17 years old and have a confirmed diagnosis of childhood asthma by a physician. In the discovery phase, prediction models for severe asthma attacks were developed using data on past asthma attacks, salivary bacterial composition, and inflammatory mediator levels in serum. The replication phase involved repeating these steps within the U‐BIOPRED dataset, adapting the analysis to the available data, and employing the same approach as the discovery phase. All centers obtained approval from the local medical ethics committee, and written informed consents were provided by parents/carer and/or recruited children.

### 2.2 Participants

The SysPharmPediA cohort included children with moderate‐to‐severe asthma who were being treated at GINA step 3 or higher [34]. Nearly all children (96%) in this cohort were followed prospectively for 12 months from their inclusion date. During the follow‐up period, severe asthma attacks, defined as events requiring systemic corticosteroids for three or more days or resulting in hospitalization or emergency room visits for asthma treated with systemic corticosteroids [35], were recorded (referred to as future asthma attacks). Based on their experience during this follow‐up, children were categorized as “at risk” if they experienced at least one severe asthma attack, or “no‐risk” if they did not. The U‐BIOPRED cohort consisted of children with mild‐to‐severe asthma, with only those with severe asthma being followed prospectively and similarly classified according to the occurrence of severe asthma attacks during this 12‐month period.

### 2.3 Sample Collection and Omics Analysis

At the study inclusion visit, 143 samples of saliva and 125 oropharyngeal (throat) swabs were gathered from SysPharmPediA and U‐BIOPRED children, respectively. The process of preparing the samples, conducting sequencing, and processing the reads is described in detail in Supporting Information. In summary, the V3‐V4 hypervariable regions of the 16S rRNA gene were amplified and sequenced using the MiSeq v3 2 × 300 bp (Illumina) platform. Subsequently, the reads underwent quality control and were organized into amplicon sequence variants (ASVs). Finally, taxonomies were assigned to these ASVs utilizing the Silva database version 138 and 132 [36] for SysPharmPediA and U‐BIOPRED, respectively.

### 2.4 Inflammatory Mediator Assays

Full details of sample collection and inflammatory mediator assays are provided in the Supporting Information. Briefly, serum samples were used to measure levels of inflammatory mediators using Luminex multiplex immunoassays (R&D Systems Inc., Minneapolis, MN). All assays were analyzed using a Bio‐Plex 200 system (Bio‐Rad, Hercules, CA, USA) [37].

### 2.5 Data and Statistical Analysis

#### 2.5.1 Microbiome Analysis

Initially, bacterial taxa were agglomerated at the genus level, and the comparison involved assessing overall diversity (alpha/beta diversity) of the samples between groups (Figures S3, S4). Subsequently, individual genera were analyzed using a differential abundance analysis (ANCOM‐BC R package version 2.0.3 [38]) (Figure S5).

#### 2.5.2 Predictive Models of Asthma Attacks

We aimed to assess the predictive utility of past asthma attacks, saliva microbiome composition, and inflammatory mediator levels in serum for predicting asthma attacks in the future. Classification models were constructed using the random forest (RF) method from the randomForest R package version 4.7–1.1 [39]. Three strategies were employed: classification using each dataset individually, classification using combined datasets constructed by pairing each dataset (results reported in Table S2), and classification using a combined model that included all data sources. Initially, the discovery dataset (SysPharmPediA) was randomly divided into training (70%) and test (30%) subsets based on several considerations, including literature, the sparsity of microbiome data, and the need to maintain a balanced and meaningful distribution of groups in both subsets [40], using stratified sampling to maintain proportional class distribution. An RF model (ntree = 500 (default ntree) and default mtry, i.e., square root of the number of features) was trained on the training set using the caret R package version 4.2.3 [41]. To optimize feature selection, we applied 5‐fold cross‐validation, balancing between bias and variance in performance estimation while ensuring that each fold contains a more representative distribution of both groups, with 10 repetitions, using classification accuracy as the performance metric. This process was conducted separately for each data layer (inflammatory mediators and microbiome) to identify the most predictive features. The top‐ranked variables from each layer, those contributing most to model accuracy, were selected and used to construct the final model. This final model was then applied to the independent, blinded test set for validation [42].

The models' performances were evaluated by calculating the area under the receiver operating characteristic curves (AUROCCs) along with their corresponding 95% confidence intervals (CI) as well as sensitivities, specificities, and accuracies for which the Youden index was used to calculate the best threshold within the AUROCCs using pROC R package version 4.2.3. In the replication phase (U‐BIOPRED), leave‐one‐out cross‐validation was employed to calculate AUROCCs, providing reliable estimates of the models' predictive power and generalizability. Pairwise comparisons of ROC curves between individual models and the combined model were performed using the DeLong test. All analyses were performed using RStudio (version 2022.02.3 + 492) with R software (version 4.2.1).

### 2.6 Exploratory Analysis

- Assessing the predictive potential of fractional exhaled nitric oxide (FE_NO_) and blood eosinophil counts in predicting future asthma attacks. In the discovery phase (SysPharmPediA), individual models were developed using these variables. After training, the models were validated on the test set to evaluate their performance and generalizability.
- Exploring differences between the at risk and no‐risk groups, focusing on dietary intake (in the past day, collected through food diary [43, 44, 45]), living environment, and antibiotic use through Mann–Whitney U test for numerical, and Fisher's exact tests for categorical variables (reported in the Table S3).
- Investigating the influence of sex, OCS, and biologics use on the taxa and inflammatory mediators of the combined model through Mann–Whitney U test (reported in the Table S4).

## 3 Results

### 3.1 Participant Characteristics

**FIGURE 1:**
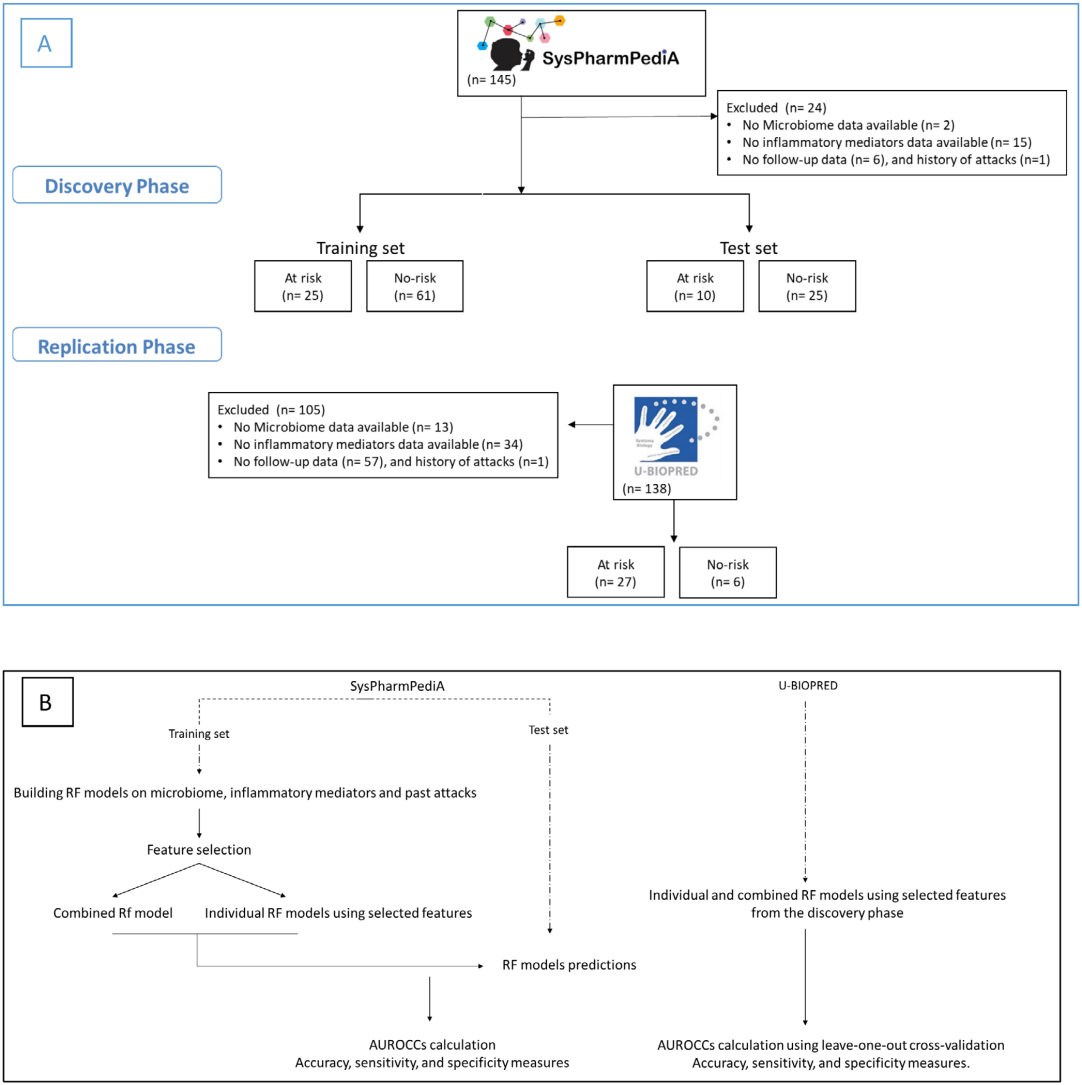
Flowchart of the participants enrolled in the study (panel A) and analysis plan (panel B). AUROCC, Area under the receiver operating characteristic curve; RF, Random forest; SysPharmPediA, Systems Pharmacology Approach to Uncontrolled Pediatric Asthma; U‐BIOPRED, Unbiased Biomarkers for the Prediction of Respiratory Disease Outcomes.

A flowchart of participants' enrollment and data analysis is depicted in Figure 1. Of the 143 participants in the SysPharmPediA cohort who provided a saliva sample, 128 had inflammatory mediator levels in serum available. Among these, 121 participants had complete data on past asthma attacks and follow‐up information (future attacks). Of the 138 school‐aged children in the U‐BIOPRED cohort, 43 with severe asthma were followed up. Among these, 33 had complete data on oropharyngeal microbiome composition, serum inflammatory mediator levels, and both past and future asthma attacks. The baseline characteristics of the participants are summarized in Table 1.

**TABLE 1:** Demographic data and baseline characteristics of the study population.

	SysPharmPediA			U‐BIOPRED		
	At risk (*n* = 35)	No‐risk (*n* = 86)	*p*	At risk (*n* = 27)	No‐risk (*n* = 6)	*p*
Age in years						
Median (IQR)	11.2 (9.8, 13.2)	12.1 (9.7, 14.1)	0.439	13.0 (11.0, 14.0)	11.0 (11.0, 11.8)	0.09
Min‐max	7.2–17	6–17.4		6–17	6–13	
Sex, (%)						
Female	21 (60.0)	30 (34.9)	0.015	16 (59.3)	4 (66.7)	1.00
BMI z‐score						
Median (IQR)	0.5 (−0.4, 1.4)	0.5 (−0.3, 1.2)	0.55	1.9 (0.5, 2.4)	0.5 (0.4, 1.4)	0.11
(Second‐hand) smoking, (%)						
Present	10/34^b^ (29.4)	24/82 (29.3)	1.00	4/25 (16)	3/5 (60.0)	0.68
Ethnicity, (%)						
Caucasian	27 (77.1)	68 (79.1)	0.811	24 (88.9)	3 (50.0)	0.58
Country of inclusion, (%)						
Netherlands	8 (22.9)	16 (18.6)	0.056	6 (22.2)	1 (16.7)	0.35
Slovenia	5 (14.3)	15 (17.4)		—	—	
Spain	19 (54.3)	30 (34.9)		—	—	
Germany	3 (8.6)	25 (29.1)		—	—	
United Kingdom	—	—		18 (66.7)	3 (50.0)	
Sweden	—	—		3 (11.1)	2 (33.3)	
(Childhood) Asthma control test^a^						
Median (IQR)	20.0 (16.5, 23.0)	*n* = 83; 23.0 (21.0, 25.0)	< 0.001	13.0 (9.5, 18.0)	18.0 (14.8, 19.8)	0.20
Allergy characteristics						
Allergic rhinitis, (%)	26/34 (76.5)	62/81 (76.5)	1.00	17 (63.0)	4 (66.7)	1.00
Atopic sensitization^c^, (%)	29/32 (90.6)	76/84 (90.5)	1.00	25 (92.6)	6 (100.0)	1.00
Whole‐blood cellular counts						
Eosinophil percent Median (IQR)	*n* = 33^b^; 6.6 (4.0, 9.0)	*n* = 78; 5.3 (3.1, 8.5)	0.252	*n* = 9; 5.3 (4.3, 10.2)	*n* = 3; 7.3 (6.9, 13.5)	0.48
Eosinophil counts (cells/μL) Median (IQR)	*n* = 33; 0.47 (0.24, 0.83)	*n* = 78; 0.37 (0.22, 0.61)	0.11	*n* = 9; 0.4 (0.2, 0.8)	*n* = 3; 0.31 (0.30, 0.65)	1.00
Current asthma medication, (%)						
ICS	35 (100.0)	86 (100.0)	NA	27 (100.0)	6 (100.0)	NA
SABA	33/34 (97.1)	76/84 (90.5)	0.44	27 (100.0)	6 (100.0)	NA
LABA	31/33 (93.9)	81 (94.2)	1.00	27 (100.0)	6 (100.0)	NA
OCS (maintenance)	3 (8.6)	1 (1.2)	0.20	6 (22.2)	0 (0.0)	0.56
LTRA	5/33 (15.2)	15/75 (20.0)	0.79	21 (77.8)	3 (50.0)	0.31
Biologics	4/34 (11.8)	6/81 (7.4)	0.48	4 (14.8)	0 (0.0)	1.00
Antibiotics use, (%)						
Previous use	6 (17.1)	12 (14.0)	0.66	12 (44.4)	0 (0.0)	0.07
Spirometry % predicted, median (IQR)						
FEV_1_ pre‐bronchodilator	93.6 (81.7, 100.9)	*n* = 85; 92.6 (82.9, 103.3)	0.85	*n* = 26; 87.6 (70.6, 104.6)	*n* = 5; 68.8 (58.3, 83.6)	0.17
FEV_1_ post‐bronchodilator	99.1 (91.1, 105.6)	*n* = 84; 99.0 (89.6, 109.4)	0.75	104.0 (90.4, 113.4)	92.0 (88.4, 101.3)	0.22
FEF 25–75				69.2 (44.4, 90.9)	44.3 (33.0, 72.4)	0.25

Abbreviations: BMI, body mass index; FEF25‐75, forced mid‐expiratory flow; FEV_1_, forced expiratory volume in one second; ICS, Inhaled corticosteroids; IQR, interquartile range; LABA, long‐acting beta‐agonists; LTRA, leukotriene receptor antagonists; OCS, oral corticosteroids; SABA, short acting beta‐agonists; SysPharmPediA, Systems Pharmacology Approach to Uncontrolled Pediatric Asthma; U‐BIOPRED, Unbiased Biomarkers for the Prediction of Respiratory Disease Outcomes.

^a^ A Childhood Asthma Control Test score > 19 indicates well‐controlled school‐age asthma.

^b^ The definition of atopy in U‐BIOPRED was based on a skin‐prick test at the patient visit and in SysPharmPediA on patients' histories (medical files).

^c^ Sample size is indicated when missing data are present.

### 3.2 Predictive Models for Future Asthma Attacks in SysPharmPediA Cohort

#### 3.2.1 Past Attacks as Predictor of Future Asthma Attacks

RF modeling using past asthma attacks resulted in AUROCC of 0.74 (95% CI: 0.62–0.85) for the training set and AUROCC of 0.72 (95% CI: 0.53–0.91) for the test set.

#### 3.2.2 Saliva Bacterial Composition as Predictor of Future Asthma Attacks

The RF model, with six bacteria genera—*Cardiobacterium*, *Rothia, Capnocytophaga*, *Gemella*, *Corynebacterium*, and *Kingella*—achieved the highest accuracy. The model resulted in an AUROCC of 0.69 (95% CI: 0.57–0.81) for the training set and an AUROCC of 0.68 (95% CI: 0.51–0.86) for the test set.

#### 3.2.3 Inflammatory Mediator Serum Levels as Predictors of Future Asthma Attacks

The RF model, utilizing six inflammatory mediators—tissue inhibitor of metalloproteinase (TIMP)‐4, vascular endothelial growth factor (VEGF), monocyte inflammatory protein (MIP)‐3β, receptor for advanced glycation end products (RAGE), TIMP‐1, and matrix metalloproteinase (MMP)‐9—demonstrated the highest accuracy. It achieved an AUROCC of 0.72 (95% CI: 0.59–0.84) for the training set and an AUROCC of 0.70 (95% CI: 0.52–0.88) for the test set.

#### 3.2.4 Combined Prediction Model of Microbiome, Inflammatory Mediator Levels, and Past Attacks

**FIGURE 2:**
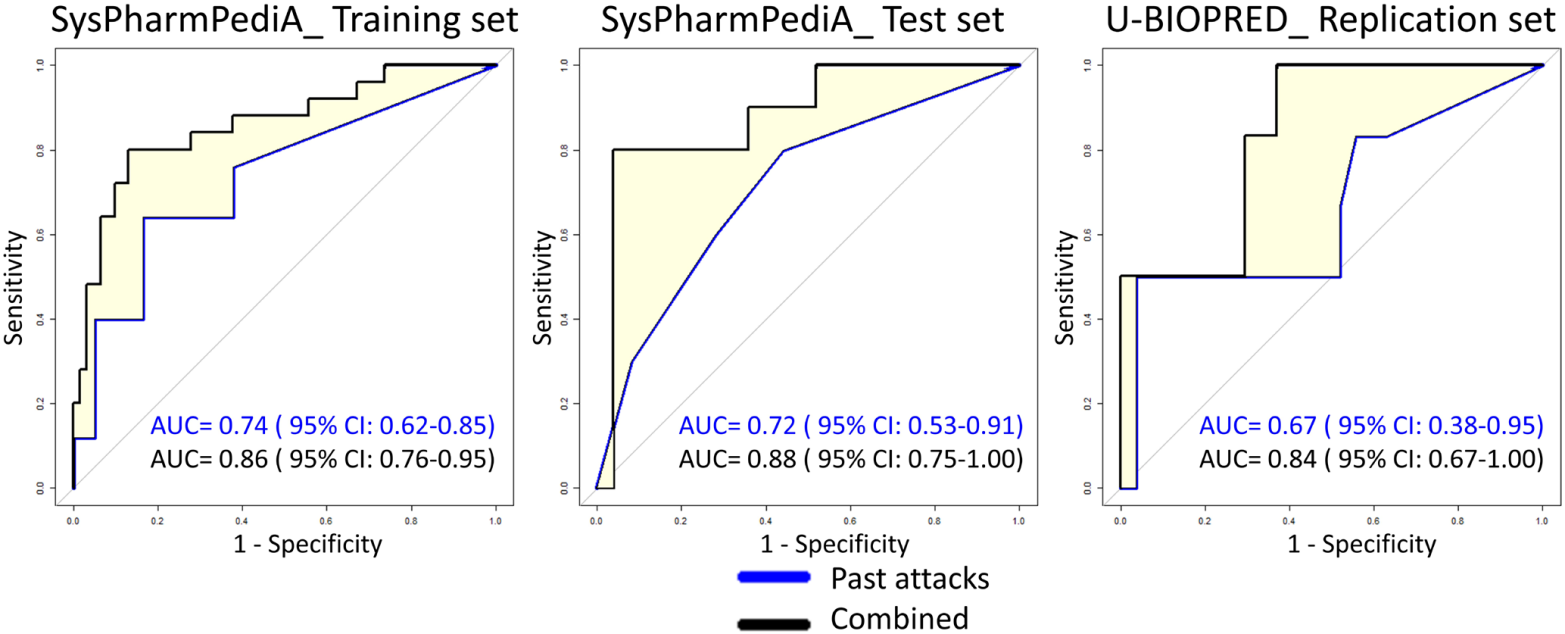
Receiver operating characteristic curves of prediction models of asthma attacks based on a model including only past asthma attacks (past attacks) and the combined model of past asthma attacks, serum inflammatory mediators, and salivary bacteria (combined). The yellow highlight shows the significant improvement in predictive performance of the combined model over the past attacks model. AUC, Area under the curve, CI, Confidence interval; SysPharmPediA: Systems Pharmacology Approach to Uncontrolled Pediatric Asthma; U‐BIOPRED: Unbiased Biomarkers for the Prediction of Respiratory Disease Outcomes.

The RF model incorporating seven features (variables)—past asthma attacks, *Capnocytophaga*, *Corynebacterium*, *Cardiobacterium*, TIMP‐4, VEGF, and MIP‐3β—demonstrated the highest accuracy. It achieved an AUROCC of 0.86 (95% CI: 0.77–0.95) for the training set and an AUROCC of 0.88 (95% CI: 0.75–1.00) for the test set (Figure 2). This suggests that combining data on past asthma attacks, serum inflammatory mediators, and the bacterial genera provides complementary information from each data layer in relation to the risk of future attacks.

### 3.3 Replication Outcomes in Subset of Children With Severe Asthma From U‐BIOPRED Cohort

Among the three inflammatory mediators identified in the discovery phase, only VEGF measured in serum samples was available in the U‐BIOPRED (Figures S1, S2). Consequently, the analysis was adapted accordingly, and the results using the same discovery approach are as follows. RF modeling using past asthma attacks resulted in an AUROCC of 0.67 (95% CI: 0.39–0.96). The microbiome model achieved an AUROCC of 0.66 (95% CI: 0.45–0.87), and the inflammatory mediator model produced an AUROCC of 0.65 (95% CI: 0.45–0.85). Ultimately, the combined model, which incorporated five features—past asthma attacks, *Capnocytophaga*, *Corynebacterium*, *Cardiobacterium*, and VEGF—resulted in an AUROCC of 0.84 (95% CI: 0.67–1.00). A summary of all models and their performance measures is provided in Table 2. The DeLong test revealed a statistically significant improvement in AUROCCs of the combined model over the past attacks model across the training, test, and replication sets (Table 3).

**TABLE 2:** Summary of performance metrics of classification models.

Models	SysPharmPediA_Training set				SysPharmPediA_Test set				U‐BIOPRED_Replication set			
	AUC (95% CI)	Sensitivity	Specificity	Accuracy	AUC (95% CI)	Sensitivity	Specificity	Accuracy	AUC (95% CI)	Sensitivity	Specificity	Accuracy
Past attacks	0.74 (0.62–0.85)	0.64	0.84	0.78	0.72 (0.53–0.91)	0.80	0.56	0.63	0.67 (0.39–0.96)	0.50	0.96	0.88
Microbiome	0.69 (0.57–0.81)	0.76	0.62	0.66	0.68 (0.51–0.86)	0.90	0.60	0.66	0.66 (0.45–0.87)	1.00	0.48	0.58
Inflammatory mediators	0.72 (0.59–0.84)	0.52	0.90	0.79	0.70 (0.52–0.88)	1.00	0.4	0.57	0.65 (0.45–0.85)	0.83	0.59	0.64
Combined	0.86 (0.77–0.95)	0.80	0.87	0.85	0.88 (0.75–1.00)	0.80	0.96	0.91	0.84 (0.67–1.00)	1.00	0.63	0.70

Abbreviations: AUC, Area Under the Curve; CI, Confidence Interval; SysPharmPediA, Systems Pharmacology Approach to Uncontrolled Pediatric Asthma; U‐BIOPRED, Unbiased Biomarkers for the Prediction of Respiratory Disease Outcomes.

**TABLE 3:** Pairwise comparison of area under the curve using DeLong test, (*α* = 0.05).

Models	SysPharmPediA_Training set	SysPharmPediA_Test set	U‐BIOPRED_Replication set
Past attacks vs. combined	*p*‐value = **0.037**	*p*‐value = **0.035**	*p*‐value = **0.045**
Microbiome vs. combined	*p*‐value = **0.015**	*p*‐value = 0.127	*p*‐value = 0.108
Inflammatory mediators vs. combined	*p*‐value = **0.027**	*p*‐value = 0.082	*p*‐value = 0.158

*Note:* Statistically significant results are provided in bold.

### 3.4 Exploratory Analysis

- Out of the 86 participants in the training set, complete data on FE_NO_ and blood eosinophil counts were available for 73 and 79 participants, respectively. The results of the models are summarized in Table 4.
- There was no significant difference with regard to dietary intake, living environment, and antibiotic use between the at risk and no‐risk groups (Table S3).
- Statistical tests did not reveal any significant influence of sex, OCS, and biologics use on the taxa and inflammatory mediators of the combined model (Table S4).

**TABLE 4:** Summary of model outcomes based on FE_NO_ and blood eosinophil counts.

Models	SysPharmPediA_Training set	SysPharmPediA_Test set
	AUC (95% CI)	AUC (95% CI)
FE_NO_	0.69 (0.56–0.81)	0.51 (0.28–0.75)
FE_NO_ category^a^	0.57 (0.45–0.68)	0.53 (0.34–0.73)
Blood eosinophil count	0.57 (0.40–0.73)	0.57 (0.36–0.78)
Blood eosinophil count category^b^	0.62 (0.53–0.72)	0.59 (0.23–0.60)

^a^ Based on FE_NO_ levels cutoff (< 20 ppb vs. ≥ 20 ppb).

^b^ Based on blood eosinophil count cutoff (< 300, ≥ 300 cells/μL).

## 4 Discussion

In this study, we developed predictive models for asthma attacks using historical data on asthma attacks, salivary bacteria composition, and serum inflammatory mediator levels. Subsequently, by integrating these profiles, we built a comprehensive predictive model in children with moderate‐to‐severe asthma (SysPharmPediA). This model showed a substantial improvement in predictive accuracy compared to the individual models; meaning it might better inform physicians when assessing the risk of future asthma attacks in a child, potentially enabling more informed clinical decisions regarding preventive interventions. We then replicated the adapted models in a subset of children with severe asthma from the U‐BIOPRED cohort, demonstrating the complementary effect of combining baseline oral bacteria data and systemic immune responses with past attacks information for predicting the risk of future asthma attacks.

There is a scarcity of multivariable prediction models for pediatric asthma attacks that incorporate both epidemiological/clinical data and objective biomarkers. A recent systematic review and meta‐analysis identified 17 prediction models from seven pediatric studies, with pooled AUCs of 0.67 for emergency department visits and 0.79 for hospitalizations. However, these models generally suffered from key limitations; they were predominantly retrospective, lacked external validation, and often relied on administrative or electronic health record data with inconsistent definitions of asthma attacks, with few incorporating biological markers or undergoing external validation [46]. Similarly, Perez‐Garcia et al. developed a predictive model for asthma attacks in children that incorporated 15 features, including two clinical variables (asthma control and asthma severity), two single‐nucleotide polymorphisms (rs279728 and rs6467778), and 11 bacterial genera from the salivary microbiome [11]. While their model utilized data on past asthma attacks, recognized as a good predictor of future events, it was not based on actual prospective data of future attacks. Their model achieved an average AUC, sensitivity, and specificity of 0.80, 0.59, and 0.85, respectively. In comparison, our study developed a prediction model based on prospectively collected data from children who subsequently experienced asthma attacks. This model represents a significant improvement in sensitivity (0.80 vs. 0.59) without compromising specificity, demonstrating its enhanced ability to accurately identify at‐risk individuals. While these models have shown moderate to good performance, they are often derived from isolated datasets, use inconsistent definitions of asthma attacks, and lack clinical interpretability. These limitations hinder their utility in real‐world settings. As such, there remains a critical need for a more robust, generalizable prediction model, one that can reliably identify children at risk of asthma attacks and offer practical relevance for use in clinical decision‐making.

Moreover, a deeper understanding of the underlying factors contributing to the attacks risk is essential for tailoring prevention strategies to individual patients. Growing recognition and evidence from microbiome studies have highlighted the significant role of bacterial dysbiosis, changes in the structure, quantity, and variety of bacteria, in increasing the host's susceptibility to infection and contributing to the development or worsening of atopy, wheeze, and asthma in children, which may stem from irregular inflammatory responses [13, 33, 47].

The interplay between the bacteria and the immune system is critical, as alterations in the bacterial composition can disrupt immune homeostasis, leading to exaggerated inflammatory responses. The presence of certain bacteria in the airways is associated with worse asthma control and increased type 2 (T2) and/or non‐T2 inflammations [48]. T2 inflammation is characterized by elevated levels of eosinophils and specific inflammatory mediators, which attract eosinophils to the site of inflammation and contribute to bronchoconstriction and mucus production, driving asthma attacks [21, 49]. Bacterial products (metabolites) can also modulate immune responses, promoting regulatory T cell (Treg) function and reducing T2 inflammation [21]. Overall, the complex interaction between the microbiome and the immune system underscores the importance of maintaining a balanced microbial environment to potentially mitigate asthma attacks. To better understand these multifaceted relationships, we applied a machine learning approach capable of detecting non‐linear interactions and patterns not apparent through conventional analyses. While such models are often considered “black boxes”, they are valuable in uncovering subtle, multivariate signals that reflect the underlying biological complexity [50, 51], particularly when studying systems as intricate and interconnected as the microbiome and immune response.

Our findings showed that, with the exception of *Capnocytophaga*, all other predictive bacteria were more abundant in participants who did not experience an attack during the follow‐up period (Figure S1). This aligns with previous research demonstrating increased levels of *Capnocytophaga* in the sputum of children experiencing asthma attacks, and it was positively correlated with MIP‐1β in the sputum, a mediator involved in eosinophil recruitment [47]. Notably, two remaining bacteria in the combined model, *Cardiobacterium* and *Corynebacterium*, were previously associated with positive health outcomes [52, 53, 54, 55]. *Corynebacterium* can impede *Streptococcus's* growth in vitro, a bacterium associated with recurrent asthma attacks in wheezing and childhood asthma [33], by secreting antibacterial substances [56]. Higher levels of *Cardiobacterium* were found in bronchoalveolar lavage fluid of non‐allergic children, and they were positively correlated with neutrophil count [57].

Among the inflammatory mediators in serum, TIMP‐4, VEGF, and MIP‐3β remained in the final predictive model (Figure S2). TIMPs regulate the extracellular matrix, thereby contributing to airway remodeling [58]. VEGF is a key mediator of angiogenesis and vascular permeability, with elevated levels linked to increased vascular leakage and inflammation in asthmatic airways, symptoms, and attack frequency [59]. MIP‐3β is a chemokine that recruits dendritic cells and lymphocytes to inflammation sites, amplifying immune responses and contributing to ongoing airway inflammation and remodeling [60]. These mediators share common pathways in promoting inflammation and tissue remodeling, which are crucial in the pathophysiology of severe asthma. The interplay of TIMP‐4, VEGF, and MIP‐3β can lead to a vicious cycle of inflammation and structural changes in the airways, increasing the likelihood of severe asthma attacks.

Bacterial components and metabolites such as lipopolysaccharides (LPS), short‐chain fatty acids (SCFAs), hydrogen sulfide (H₂S), and ammonia, originating from *Capnocytophaga*, *Corynebacterium*, and *Cardiobacterium*, may influence inflammation and tissue remodeling in asthma [21, 61, 62]. LPS and hydrogen sulfide activate pathways that lead to the production of VEGF [63, 64], while SCFAs modulate MMP activity, indirectly regulating TIMP‐4 to balance tissue degradation. Ammonia‐induced tissue damage further promotes MMP activity and TIMP‐4 upregulation to maintain tissue integrity [65, 66]. Additionally, lipoteichoic acids (LTA) activate immune responses, leading to the release of MIP‐3β [67]. These interactions may play a role in driving inflammation and contributing to the progression of asthma. Further mechanistic studies are essential to elucidate the precise roles of these bacterial components and metabolites in asthma pathogenesis.

Accurate and early identification of asthma attacks risk, particularly in children, remains a critical challenge for timely intervention and effective management. Notably, children with persistent asthma and at least one severe attack in the previous year have been shown to have a 2–2.5‐fold increased risk of subsequent severe attacks, even when on controller therapy [68, 69, 70]. While a history of previous attacks provides valuable insights, likely reflecting the cumulative impact of various underlying risk factors such as airway hyperresponsiveness, environmental exposures, medication adherence, and comorbidities [46, 71], this approach alone may not fully capture the complexity of future risk. Integrating objective, data‐driven markers alongside clinical history could significantly enhance predictive accuracy.

This study highlights the potential of objective minimally invasive measures to address this need. The integration of salivary bacterial composition and inflammatory profiles in serum showed promise for improving predictive accuracy and may offer insights into the mechanisms underlying asthma attacks. Compared to conventional biomarkers like FE_NO_ and blood eosinophils count, which showed poor predictive performance in this study, the combined model demonstrated superior predictive capability, offering a more robust tool for identifying pediatrics at risk of future asthma attacks. Notably, while a history of past asthma attacks emerged as a strong individual predictor, consistent with clinical expectations, our results show that incorporating microbiome and inflammatory mediator data significantly enhances model performance. The combined model consistently outperformed models based on single data types, underscoring the added value of a multi‐dimensional, systems‐level approach. These findings suggest that future therapeutic strategies could potentially involve modulating specific oral bacterial taxa and systemic inflammatory mediators. In the future, clinicians may find value in exploring the use of targeted probiotics, prebiotics, synbiotics, or antibiotic treatments to potentially maintain or restore a healthy oral microbiome and immune balance, ultimately leading to a reduction in asthma attacks.

The non‐invasive nature of saliva sampling makes it particularly suitable for the pediatric population, as it is less distressing and easier to collect compared to other methods. While comprehensive microbiome analysis can be costly and pose logistical challenges, focusing on specific bacterial taxa associated with asthma risk could make testing more feasible and affordable. By using a single saliva sample, it is possible to assess both bacterial composition and inflammatory mediator levels simultaneously. As advancements are made in identifying key bacterial profiles and associated inflammatory markers, more efficient, rapid, and cost‐effective methods can be developed, paving the way for the integration of microbiome‐based assessments into routine asthma management. Future studies should investigate the feasibility of analyzing a single saliva sample for specific bacterial taxa and inflammatory markers to predict asthma attack risk. Additionally, future research should investigate whether modulating specific bacteria using probiotics, prebiotics, or synbiotics could help prevent asthma attacks.

This study has several strengths. A key strength lies in its expansive European recruitment, encompassing two well‐characterized cohorts: SysPharmPediA and U‐BIOPRED, which span six distinct countries and multiple ethnic groups. This multinational and multicenter design enhances the generalizability of the findings to a broader population. This is particularly relevant when investigating the microbiome, as it can be influenced by the environment, dietary practices, and ethnic background. Furthermore, we adhered to the TRIPOD guidelines to ensure standardized methodologies, increased data quality, and greater transparency and clarity [72]. Finally, the study strengthens scientific rigor by replicating the results in an independent cohort. The findings from the discovery phase were replicated using the available data, although some adaptations were necessary. Specifically, a different microbiome compartment was analyzed in U‐BIOPRED (throat swab) which, while distinct from saliva samples, shares similar bacterial profiles and only serum VEGF was available among the inflammatory markers. Despite these adaptations, the core aspects of the models were preserved. These modifications inherently introduced potential variations that can influence the results. Nonetheless, the replicated models demonstrated consistent and improved predictive performance, specifically in the combined model. This suggests promise for the approach of using concomitant salivary microbiome and inflammatory mediators to predict the risk of future attacks.

The study has several limitations that need to be acknowledged. Its observational design allows identification of association but does not establish causality. Additionally, information regarding over‐the‐counter medications, pre(pro)‐biotic consumption, and oral health was not available; therefore, we were not able to rule out potential bias from some unmeasured confounders. The relatively moderate sample size may increase the risk of overfitting and did not permit subgroup analyses by narrower age categories. However, we employed train‐test splits during the discovery and leave‐one‐out cross‐validation during replication phases to mitigate this risk. While our results are promising, validating them in other cohorts will solidify these findings. Lastly, the inclusion of patients from academic centers, which typically serve as referral centers for severe asthma cases, may limit the generalizability of our findings for children with mild asthma managed in primary‐care settings.

### 4.1 Further Research

Further research is needed to investigate the predictive roles of specific oral bacteria and potentially oral inflammatory mediators, as well as to explore their integration and potential correlations with various omics layers. These efforts would help to elucidate the complex interactions between the microbiome and the various components and pathways within biological systems. Furthermore, the clinical utility of the developed prediction model necessitates evaluation in a prospective longitudinal study. Monitoring oral bacterial profiles and inflammatory mediator levels at multiple time points across individual children with asthma will be critical for assessing the model's effectiveness in providing early warnings of deterioration.

## 5 Conclusion

Our findings suggest that the salivary bacterial composition and serum inflammatory mediators, while informative on their own, exhibit a complementary relationship with the history of asthma attacks in children for predicting the risk of future attacks. This highlights the potential interactions between the oral microbiome and the immune system and supports further research into these interactions, which could uncover new pathophysiological mechanisms and possibly novel therapeutic approaches for asthma.

## Author Contributions

S.S.K. performed the analysis and drafted the initial version of the manuscript. S.S.K., P.B., M.I.A.‐A., and A.H.M.Z. contributed to the design of the analysis plan. All authors contributed to the acquisition of data, interpretation of the analysis, revision, drafting, and ensuring the accuracy and integrity of the analysis. All authors provided final approval of the version to be published.

## Conflicts of Interest

J.M.B. received an honorarium from Vitakruid. I.M.A. reports support for the present study from EU funding through IMI, grants from GSK, MRC, EPSRC, and Sanofi, consultancy fees from GSK, Sanofi, and Kinaset, and payment or honoraria for lectures, presentations, manuscript writing, or educational events from AstraZeneca and Sanofi. K.F.C. has received honoraria for participating in Advisory Board meetings of Roche, Merck, Shionogi, and Rickett‐Beckinson and has also been remunerated for speaking engagements for Novartis and AZ. U.P. received the SysPharmPedia grant, co‐financed by the Ministry of Higher Education, Science and Innovation Slovenia (MVZI) (contract number C3330‐16‐500,106) and Slovenian Research Agency (research core funding No. P3‐0427 and research grant No. J3‐4497). F.S. received honoraria from Vertex Pharmaceuticals, Novartis Pharma, and payments from Vertex Pharmaceuticals to his institution and travel support from Chiesi Pharmaceuticals and Sanofi‐Aventis. B.N. is a board member and has stocks in MediTuner AB, Sweden. SuHa reported payment from Nutricia and Allergopharma. J.T. has received a speaking fee from AstraZeneca. G.R. and C.A. report support for the present study from EU funding through IMI. C.S.M. received lecture fees from Sanofi, GSK, and AstraZeneca and reports grants paid to the institution from National Institute for Health research, Asthma and lung UK, Moulton charitable Foundation, Innovate UK, and support from Sanofi to attend the ERS conference. R.L. reports support paid to the institution from Eurostar EU funding, Foresee, and Amsterdam UMC Foundation. He received an honorarium from Pfizer. Additionally, he received support for attending meetings from the European Society for Clinical Investigation (ESCI) and served in an unpaid leadership role for ESCI Trust as secretary/treasurer. He also served on an advisory board for Sanofi. R.D. reports consulting fees from Synairgen plc; GSK, ZenasBio, Celltrion, and has shares in Synairgen plc. S.E.D. reports grants from the EU, Swedish MRC, Swedish Heart Lung Foundation, Swedish Strategic Research Foundation, Stockholm County Council Research Funds, AstraZeneca, Cayman Chemical, GSK, and Sanofi. He received consulting fees from AstraZeneca, Affibody, GSK, and Sanofi, and payments for lectures from AstraZeneca and GSK. J.S. reports grants from ERC Consolidator Grant (EU), DFF—FSS Research Project 1 (Danish), Novo Nordisk Foundation, Clinical and Translational Medicine (Danish), Fabrikant Vilhelm Pedersen og Hustrus Legat (Danish). M.K. received funding from German Ministry of Education and Research, European Research Council, European Union, and Bavarian Ministry of Health. He received consulting fees from AstraZeneca. He also received honoraria from EAACI, Novartis, Nutricia, and Pari. K.B. reports consulting fees from Sanofi and AstraZeneca, lecture honoraria from Boehringer Ingelheim, and advisory board participation with ALK‐Abelló Nordic, outside the submitted work. J.W.D. reports grants from Abbvie and Boehringer Ingelheim. L.F. reports a grant from NIHR HTA paid to the institution and received consulting fees from AstraZeneca, Sanofi, GSK, Regeneron paid to the institution and reports honoraria from AstraZeneca and Sanofi paid to the institution. M.G. received the SysPharmPedia grant, co‐financed by the Ministry of Education, Science and Sport Slovenia (MIZS) (contract number C3330‐16‐500,106) and funded by the Slovenian Research Agency (research core funding No. P3‐0427) and by the Ministry of Education, Science and Sport of the Republic of Slovenia, grant PERMEABLE (contract number C3330‐19‐252,012). M.P.Y. received funding from Instituto de Salud Carlos III (AC15/00015) as part of the SysPharmPediA consortium, and grants by MCIN/AEI/10.13039/501100011033, GlaxoSmithKline Spain, and CSL Berhing outside of the submitted work. A.H.M. is the PI of a public‐private consortium (P4O2 (Precision Medicine for More Oxygen)) sponsored by Health Holland involving many private partners that contribute in cash and/or in kind (AbbVie. Boehringer Ingelheim, Breathomix, Clear, Fluidda, Ortec Logiqcare, Olive, Philips, Quantib‐U, Smartfish, Clear, SODAQ, Thirona, Roche, TopMD, Novartis, RespiQ). She received unrestricted research grants from GSK and Boehringer Ingelheim and a Vertex Innovation Award grant from Vertex. She has received honoraria from Boehringer Ingelheim, GSK, and AstraZeneca. Others have no potential conflicts of interest to disclose.

## Supporting information


**Appendix S1:** all70004‐sup‐0001‐AppendixS1.docx.

**Appendix S2:** all70004‐sup‐0002‐AppendixS2.pdf.

**Appendix S3:** all70004‐sup‐0003‐AppendixS3.pdf.

## Data Availability

The data that support the findings of this study are available from the corresponding author upon reasonable request.
