# Supplementary material for: Complementary Predictors for Asthma Attack Prediction in Children: Salivary Microbiome, Serum Inflammatory Mediators, and Past Attack History
Source: Allergy. 2025 Aug 18;81(2):413–26. doi: 10.1111/all.70004 (PMC12862560; doi:10.1111/all.70004)
Supplement: Supplementary file 1 — Appendix S1: all70004‐sup‐0001‐AppendixS1.docx. [file ALL-81-413-s002.docx]

**Supplementary information**

**1 Sample preparation, sequencing and read processing**

**1.1 Sample preparation**

**1.1.1 Saliva samples**

143 saliva samples were collected in 50ml Falcon tubes and stored locally at -80°C until transferred to a centralized biobank in Regensburg, Germany. Total DNA was extracted using the QIAamp DNA mini kit (Qiagen) and the QIAcube semi-automated system (Qiagen). 200 µL of saliva was added to 200 µL of AL buffer and incubated for 10 minutes at 56°C with continuous shaking at 900 RPM. Further preparation was performed according to the manufacturer’s protocol.

**1.2 16S rRNA isolation and sequencing**

PCR amplification was performed on both saliva and fecal samples, using primers that were chosen such that only the hypervariable regions V3-V4 of 16S rRNA were amplified. The PCR primers and cycling rotations can be found in Supplementary Information 2. The samples were sequenced using the MiSeq v3 2x300 bp (Illumina) under the standard Illumina protocol. Sequencing was considered successful when 10000 reads per sample were reached. Mock communities (ZymoBIOMICS, Microbial Community DNA Standard) and negative control samples were added to assess the sequencing quality and contamination.

**1.3 Read processing**

The paired-end reads (2x300 bp) were quality controlled using FastQC and MultiQC (1, 2). Subsequently, primers were removed using Cutadapt (3). The DADA2 pipeline, described in Callahan *et al.*, was then followed as recommended (4). The complete pipeline can be found on GitHub (<https://benjjneb.github.io/dada2/tutorial.html>). In short, this pipeline works by performing quality control and consequently filtering and trimming reads, dereplicating sequences, inferring dataset-specific error rates, denoising by removing potential sequencing errors, merging paired-end reads, and constructing a sequence table. Chimeras are removed by implementing the “bimera” method. Finally, amplicon sequence variants (ASVs) were assigned taxonomies using publicly available databases. In this case, the Silva database version 138 was used for taxonomic classification (5).

**2 PCR parameters**

**2.1 PCR primers**

V3-V4 highly variable region:

Forward primer - hV3F - CCTACGGGAGGCAGCAG

Reverse primer - hV4R – GGACTACHVGGGTWTCTAAT

**2.2 PCR cycling conditions**

| Denaturation | 98°C | 30 sec | 30 cycles |
| --- | --- | --- | --- |
| Denaturation | 98°C | 9 sec |  |
| Annealing | 50°C | 60 sec |  |
| Elongation | 72°C | 90 sec |  |
| Final elongation | 72°C | 10 min |  |
| Hold | 12°C | forever |  |

**3.1 Oropharyngeal swabs and 16S gene V4 microbiome sequencing**

A single oropharyngeal (throat) swab was collected from the posterior pharyngeal wall, behind the uvula, of each subject by research assistants using dry flocked swabs (ESwab, BD, NJ,USA). The swabs were placed in a Liquid Amies transport medium and stored at -20°C for further processing. Genomic FastDNA™ SPIN Kit for Soil (MP Biomedicals, Santa Ana, CA, USA) was used for DNA extraction and DNA extracts were checked by gel electrophoresis for consistency and quality. A two-step PCR process was implemented to amplify the 16S ribosomal RNA (rRNA) by targeting the hypervariable V4 region by primers (515F: 5'-GTGCCAGCMGCCGCGGTAA and 806R: 5'-GGACTACHVGGGTWTCTAAT) and normalization for amplicon contents was performed before undergoing sequencing. Sequencing was done using the Illumina MiSeq Desktop Sequencer (Illumina Inc., CA, USA) as described previously (6) . Paired-end reads (250 bp x 2) were pooled for subsequent quality control and bioinformatics processing. The quality of the sequence reads were checked using FastQC (1) and MultiQC (2) . Primers were removed using Cutadapt (3). The dada2 pipeline was used to quantify the Amplicon Sequence Variants (ASVs) as described in detail previously (4). Briefly, the pipeline works by performing quality filtering and trimming, dereplicating sequences, learning dataset-specific error rates, denoising by removing potentially containing errors sequences, merging paired-end reads while removing mismatches to reduce errors, constructing amplicon sequence variants (ASVs), removing chimera, and running taxonomic classification of ASVs using different publicly available databases. During the trimming and filtering step of the forward and reverse sequencing reads, quartiles of the quality score distribution of nucleotide positions were inspected. Parameters for the reads filtering were used as follows; truncQ=2 (truncate reads at the first instance of a quality score less than or equal to 2), maxEE=5 (reads with higher than 5 expected errors will be discarded), and rm.phix=TRUE (discard reads that match against the phiX genom). Then, learning of the error rates was performed. The estimated error rates were in a good fit to the observed rates, and the error rates dropped with increased quality as expected (4). Afterwards, the, forward and reverse reads were merged using the default parameters in dada2 (minimum length of overlap=20 nucleotides, and maximum nucleotides mismatches=0). Subsequently, chimeric sequences were removed by implementing the Bimera method (removeBimeraDenovo function in dada2). Fourteen % of the total sequences were identified as chimera and subsequently removed from the final identified ASVs table. Finally, taxonomy of the identified ASVs was annotated using the Ribosomal Database Project (RDP) Naive Bayesian Classifier algorithm (7) implemented in dada2 using the default parameters (minimum bootstrap confidence for assigning a taxonomic level=50) against the Silva database version 132 (5). Negative extraction (sterile water) and positive (mock community, BEI Resources, NIAID, NIH) controls were added to each plate (sequencing run) as previously described (6), and checked against real samples by comparing Shannon α-diversity (using Mann-Whitney U test) and by relative abundance plots. As a sanity check, a statistical method to remove potentially contaminant sequences (decontam R package) (8) was used to filter the ASVs table (using the prevalence method) before downstream statistical analysis.

**4. Inflammatory mediator assay**

In SysPharmPediA, serum levels of IL-1β, IL-4, IL-6, IL-7, IL-8, IL-10, IL-13, IL-17A, IL-18, IL-23, IL-33, thymic stromal lymphopoietin (TSLP), matrix metalloproteinase (MMP)-1, MMP-3, MMP-9, tissue inhibitor of metalloproteinase (TIMP)-1, TIMP-2, TIMP-4, tumor necrosis factor (TNF)-α, thymus and activation-regulated chemokine (TARC), interferon (IFN)-γ-induced protein 10 (IP10), C-reactive protein (CRP), cluster of differentiation 14 (CD14), pulmonary and activation-regulated chemokine (PARC), macrophage-derived chemokine (MDC), growth-related oncogene (GRO)-α, vascular endothelial growth factor (VEGF), Periostin (OSF2), monocyte chemoattractant protein (MCP)-4, monocyte inflammatory protein (MIP)-3b, and receptor for advanced glycation end products (RAGE) (R&D systems INc, Minneapolis, MN), IL-9 and IL-22 (Invitrogen/ProcartaPLex) were measured using Luminex multiplex assays using fresh aliquots (samples were not thawed before) as described previously [1-3]. For IL-5 a high-sensitivity assay (R&D systems INc, Minneapolis, MN) was used. Samples were analyzed using a Bioplex 200 system (Bio-Rad, Hercules, CA, USA). Measurements with a bead count lower than 25 were deemed of a too low quality and excluded in the analysis. LODs for each marker are shown in Table S1. As a sensitivity analysis, all the analyses were repeated without imputation of values below the LODs. All Luminex multiplex immunoassays were performed by two expert and experienced technicians at the same day. And depending on the data of the controls add: and no inter-plate variation was observed.

In U-BIOPRED, Multiplex assays were designed from the R&D Systems Human Luminex Screening Assay (LXSAH) panel of reagents for the following 20 analytes: DPPIV, LBP, MMP-9, SHBG, Galectin-3, Lumican, Serpin E1/PAI-1 (in a 7-plex with 100-fold dilution of serum), and IL-8, VEGF, MMP-3, RAGE, CCL13/MCP-4, IL-33, C5/C5a, CHI3L1/YKL-40, CCL20/MIP-3α, CD40L, IL-18, IL-1α, α1-microglobulin (in a 13-plex with 4-fold dilution of serum), (Bio-Techne, Minneapolis, Minnesota, USA). For both the 7-plex and 13-plex QC-samples were included at three levels in serum with fit-for-purpose validation on-line. Analyses were performed according to the manufacturers’ instructions and measurements were made using the Bio-Plex^®^ 200 Luminex system (BIO-RAD, Hercules, California, USA). Each plate included a randomised mix of samples from each U-BIOPRED paediatric subject group.

Table S1. Upper and lower limit of detections (LOD) for inflammatory mediators

| **Mediator Name** | **Lower (pg/ml)** | **Upper (pg/ml)** |
| --- | --- | --- |
| IL-1β | 6.44 | 3992.49 |
| IL-4 | 6.88 | 3949.99 |
| IL-5 | 0.12 | 1710.6 |
| IL-6 | 0.43 | 1010.52 |
| IL-7 | 1.91 | 1152.18 |
| IL-8 | 0.4 | 949.99 |
| IL-9 | 0.48 | 28598.63 |
| IL-10 | 0.53 | 1029.99 |
| IL-13 | 15.96 | 115014.5 |
| IL-17 | 1.17 | 3156.54 |
| IL-18 | 4.63 | 4048.67 |
| IL-22 | 5.41 | 20055.99 |
| IL-33 | 2.23 | 3976.08 |
| TSLP | 0.23 | 701.81 |
| MMP-1 | 1.56 | 11225.5 |
| MMP-3 | 2.66 | 17879.96 |
| MMP-9 | 16.57 | 31531.46 |
| TNF-𝛼 | 0.82 | 1793.46 |
| TARC | 12.86 | 25329.43 |
| PARC | 2.07 | 3704.66 |
| GRO-𝛼 | 54.86 | 12312.6 |
| TIMP-1 | 1.86 | 10332.16 |
| TIMP-2 | 36.79 | 23905.19 |
| TIMP-4 | 0.63 | 5255.49 |
| MCP-4 | 1.21 | 1099.98 |
| CD14 | 48.29 | 103033.1 |
| VEGF | 0.23 | 2010.33 |
| MIP-3b | 0.4 | 2533.95 |
| IP10 | 0.2 | 400.86 |
| OSF2 | 560.34 | 391252.4 |
| MDC | 20.93 | 11533.07 |
| RAGE | 5.07 | 36980.92 |
| CRP | 3.52 | 26382.51 |

**4 Statistical analysis**

**4.1 Data preparation**

Microbiome counts were normalized using the cumulative sum-scaling normalization method implemented in the metagenomeSeq R package (9). This approach aims to adjust sequence counts based on the relative abundance of low-expressed features. Inflammatory mediator levels were normalized using a Z-score transformation.

**4.2 Global diversity measures**

Statistical analysis was performed in R using RStudio (10). Saliva samples were handled separately for all following analyses. The global diversity measures at a genus were assessed using the phyloseq and vegan R packages (11, 12). Differences in alpha diversity (diversity within samples) between groups were compared using the observed number of species and the Shannon index. Significance was assessed using the Wilcoxon rank-sum test. The beta diversity (diversity between samples) was compared using a principal coordinate analysis on the Bray-Curtis distance measures, with *p*-values being calculated by running a PERMANOVA model. A *p*-value below 0.05 was considered to be significant. Diversity analysis was adjusted for sex, age, country of inclusion, BMI (z-score), antibiotic use, oral corticosteroid use, inhaled corticosteroids dose (low, medium, high).

**4.3 Conventional differential abundance analysis (ANCOMBC)**

The conventional differential abundance analysis was performed using the ANCOMBC package (13). This method also performs its own internal normalisation. Taxonomies present in at least 5% of samples were included as previously described (14). The following covariates were included in the model, sex, age, country of inclusion, BMI (z-score), antibiotic use, oral corticosteroid use, inhaled corticosteroids dose (low, medium, high). A Benjamini-Hochberg correction was applied to correct for multiple testing, with an adjusted *p*-value below 0.05 considered to be significant.

**Results**

Figure S1. Boxplots showing the relative abundance at genus level that remained in the combined model


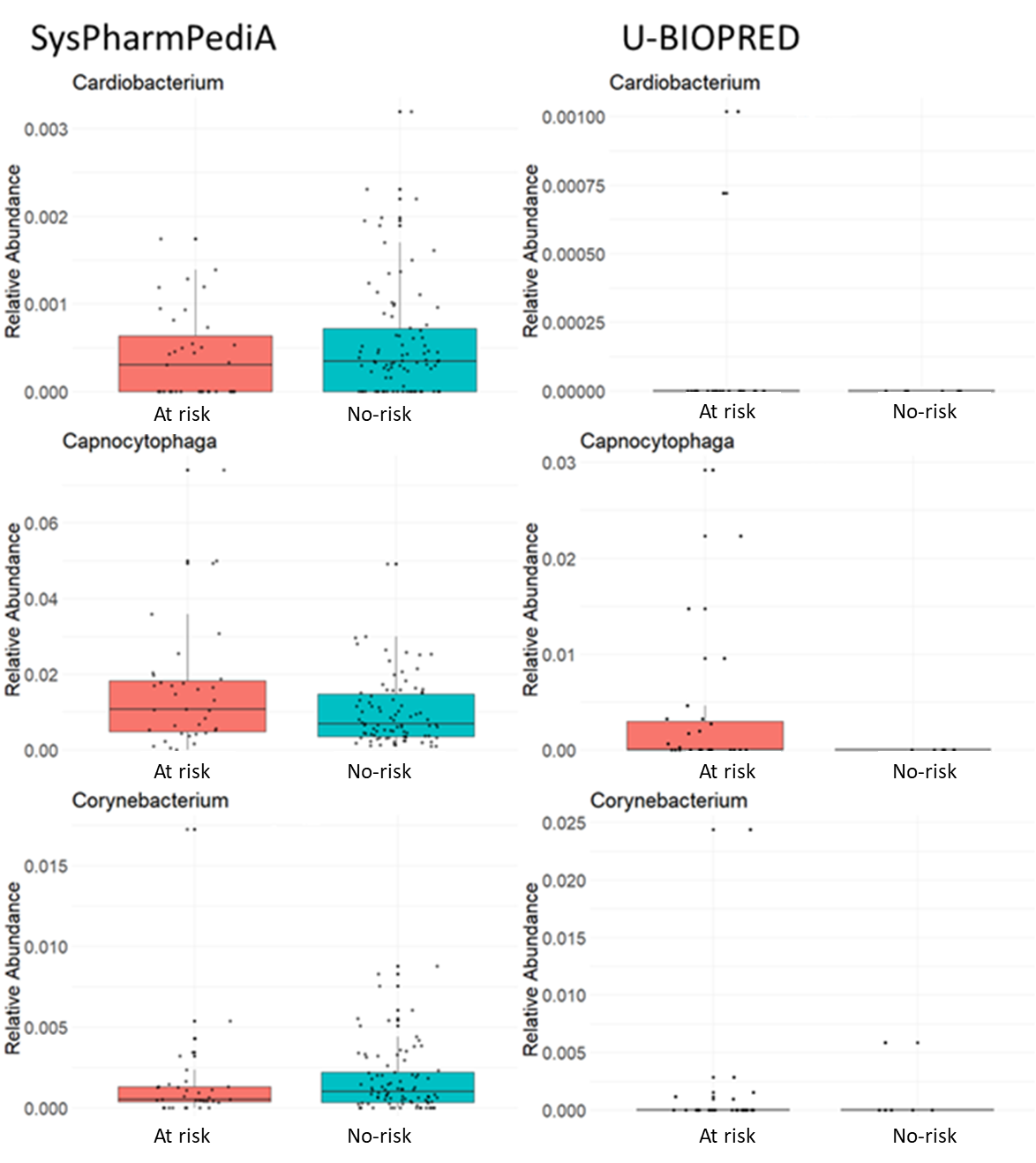


Figure S2. Violin plots showing inflammatory mediator levels that remained in the combined model


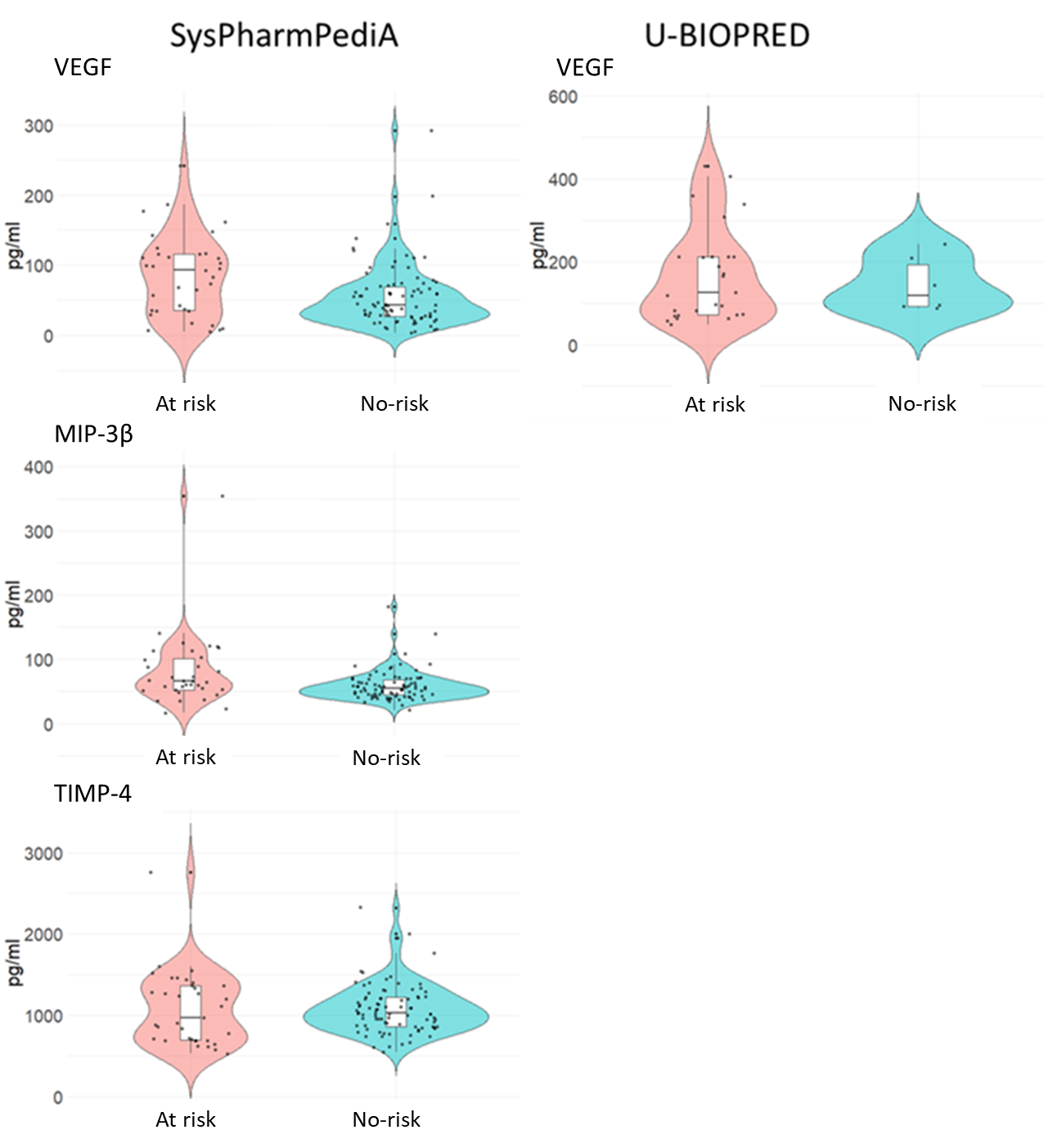


**Global diversity**

For the alpha diversity, there were no statistically significant differences between the groups, based on the number of observed unique ASV per sample (p = 0.11) or the Shannon index p = 0.83). There were significant differences with regard to beta diversity between the groups (p=0.048).

Alpha diversity


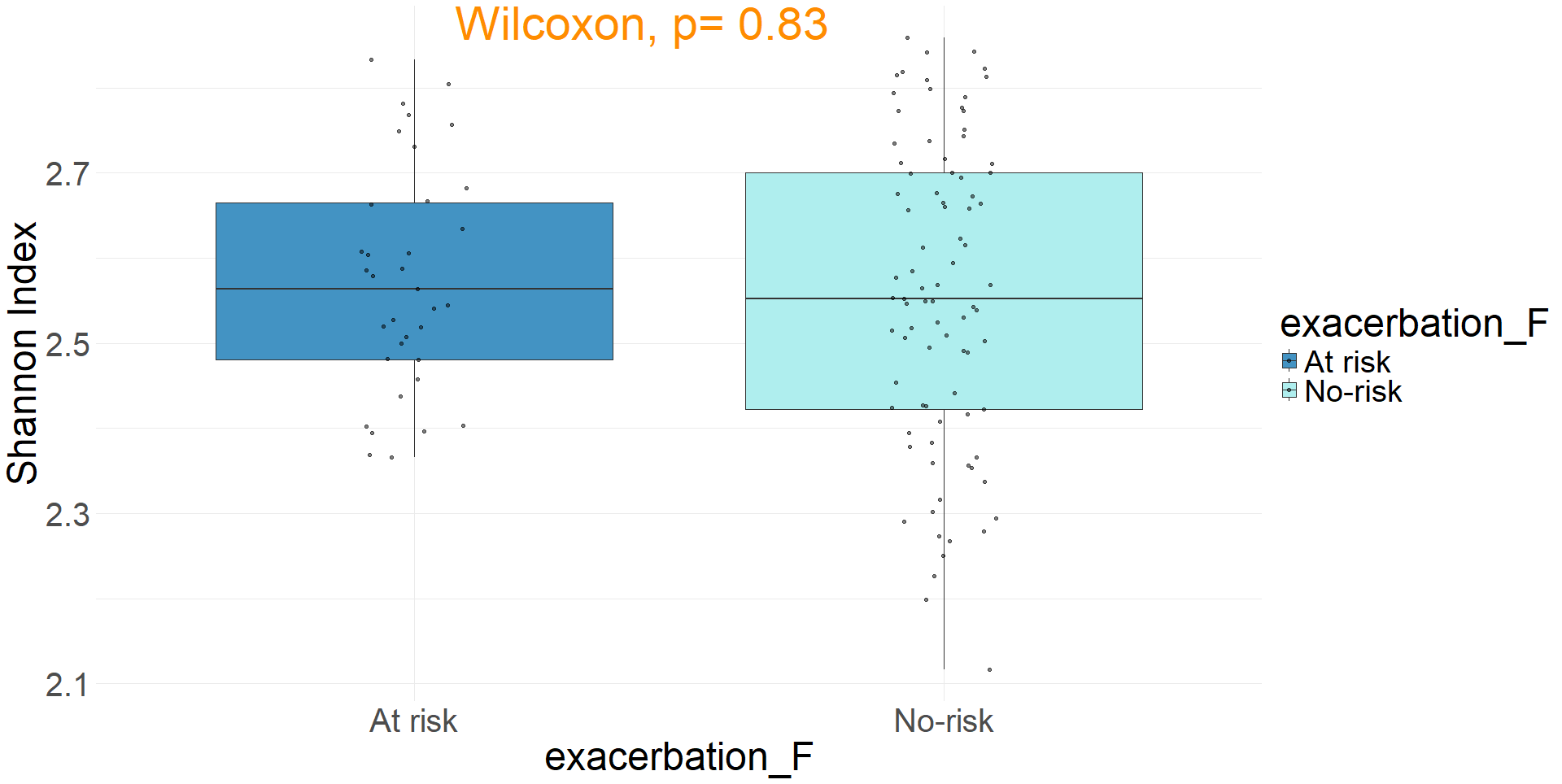


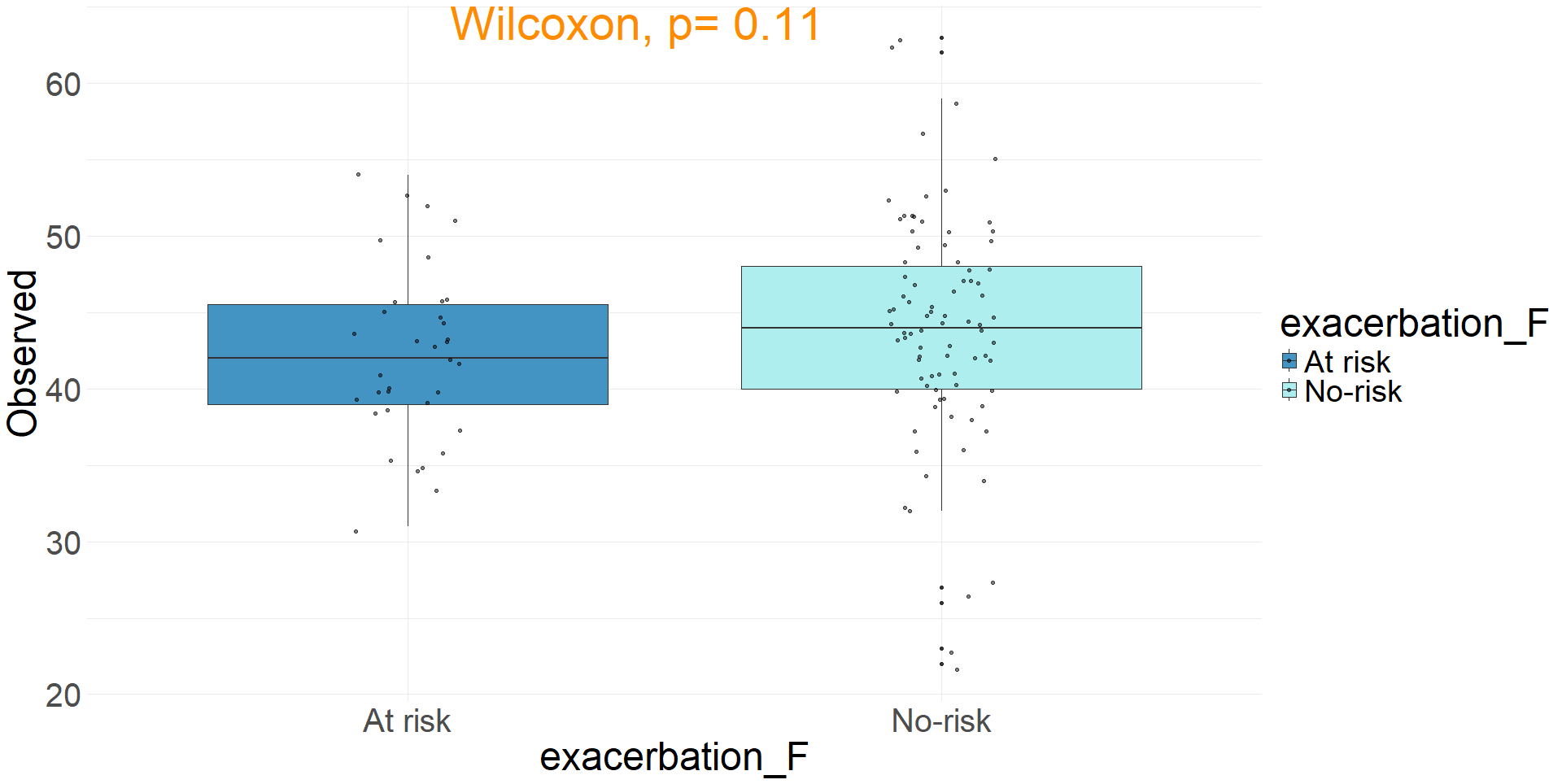


Figure S3: Alpha diversity comparing two groups using the Shannon index (A) and observed number of ASVs per sample (B). No statistical differences were found in any of the comparisons.

Beta diversity


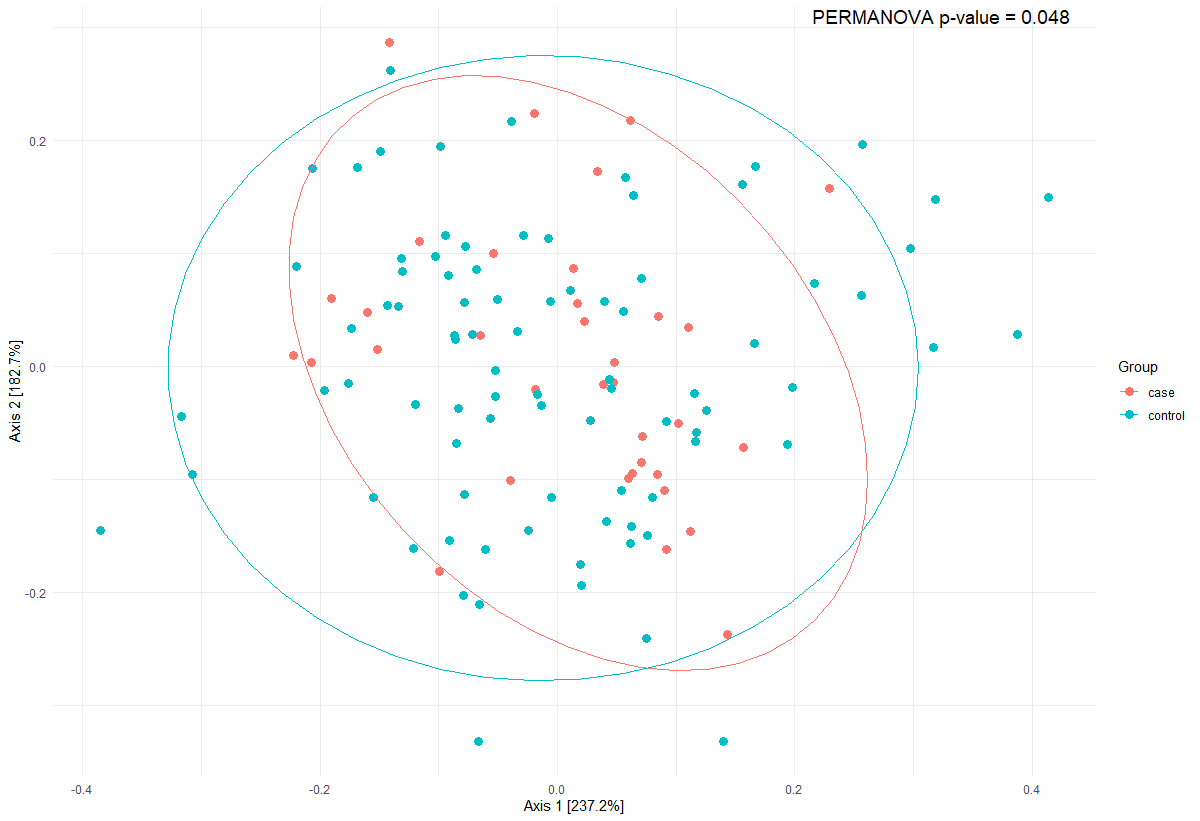


Figure S4. Beta diversity showing the groups using Principal Coordinate Analysis (PCoA) on the Bray–Curtis distance. Red dots showing at risk group and blue one representing no-risk.

**Differential abundance analysis**

After correction for sex, age, country, BMI (z-score), antibiotic use, oral corticosteroids consumption, inhaled corticosteroids dose (low, medium, high), the ANCOM-BC function revealed no statistically significant differentially abundant genera between the groups.


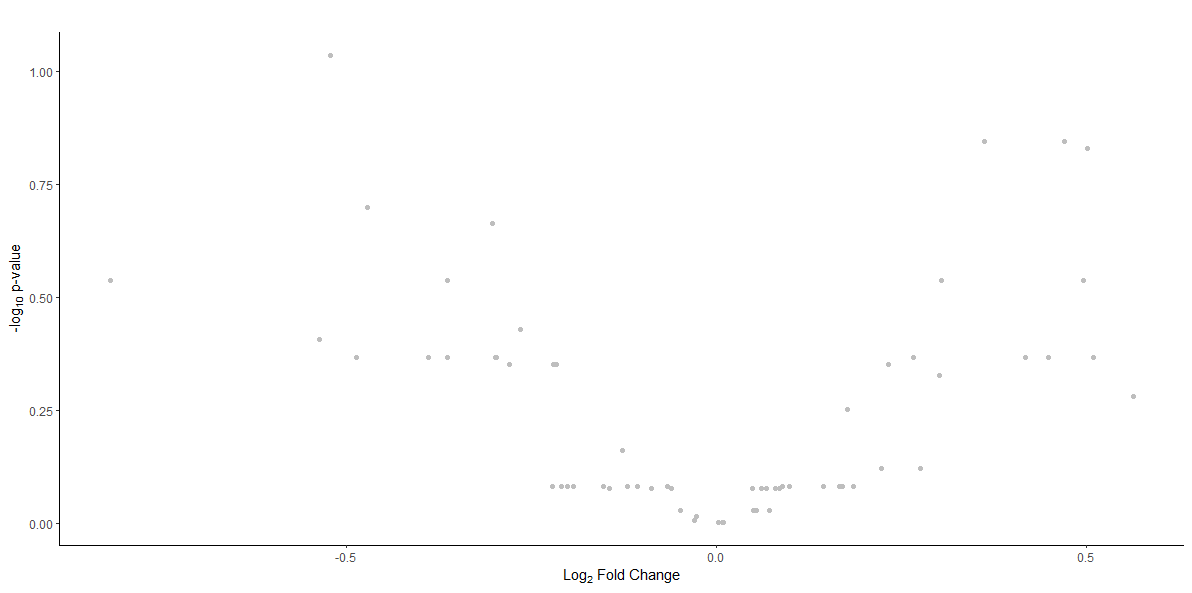


Figure S5. Volcano plot of the conventional differential abundance analysis using the ANCOM-BC function. No significantly different taxa were found in the saliva samples.

**Paired models for future asthma attacks in SysPharmPediA cohort**

Paired prediction model of microbiome and past attacks

The RF model incorporating two features (variables)—past attacks and Rothia reached highest accuracy.

Paired prediction model of inflammatory mediators and past attacks

The RF model, utilizing six inflammatory mediators_ TIMP-4, VEGF, MIP-3b, RAGE, TIMP-1, MMP-9_ and past attacks demonstrated highest accuracy.

Paired prediction model of inflammatory mediators and microbiome

The RF model, with three bacteria genera_ Capnocytophaga, Rothia, Corynebacterium and six inflammatory mediators_ TIMP-4, VEGF, MIP-3b, RAGE, TIMP-1, MMP-9 achieved the highest accuracy.

Table S2. Summary of performance metrics of paired classification models

| Models | Training set | | | | Test set | | | |
| --- | --- | --- | --- | --- | --- | --- | --- | --- |
|  | AUC (95% CI) | Sensitivity | Specificity | Accuracy | AUC (95% CI) | Sensitivity | Specificity | Accuracy |
| Past attacks + Microbiome | 0.74 (0.61-0.87) | 0.76 | 0.79 | 0.78 | 0.73 (0.51-0.96) | 0.60 | 0.96 | 0.86 |
| Past attacks + Inflammatory mediators | 0.81 (0.70-0.91) | 0.76 | 0.82 | 0.80 | 0.68 (0.62-0.94) | 0.80 | 0.76 | 0.77 |
| Microbiome + Inflammatory mediators | 0.78 (0.66-0.91) | 0.68 | 0.87 | 0.81 | 0.70 (0.50-0.90) | 0.64 | 0.92 | 0.71 |

Table S3. Comparing potential factors influencing salivary microbiome between at risk and no-risk groups in SysPharmPediA

|  | **SysPharmPediA** | | **P-value** |
| --- | --- | --- | --- |
|  | **At risk**  **(N = 35)** | **No-risk**  **(N = 86)** |  |
| **Antibiotic intake, (%)** | 6 (17.1) | 12 (14.0) | 0.66 |
| **Living environment, (%)** | | | 0.54 |
| Urban | 18/34^$^ (53.0) | 38/84 (45.2) |  |
| Rural | 16/34 (47.0) | 46/84 (54.8) |  |
|  | | | |
| **Food intake, Median [IQR]** ^#^ | **At risk**  **(N = 28)** | **No-risk**  **(N = 50)** |  |
| Energy (kcal) | 2011 [1453-2716] | 1820 [1479-2150] | 0.55 |
| Fat (g) | 68.9 [49.45-99.1] | 74 [60.33-90.08] | 0.66 |
| Saturated fat (g) | 27.9 [14.9-33.55] | 28.4 [20.85-40.04] | 0.24 |
| Protein (g) | 106.9 [64.7-121.55] | 85.05 [63.6-110.9] | 0.25 |
| Carbohydrates (g) | 218 [152.5-293.45] | 195 [156.1-238.38] | 0.43 |
| Fibers (g) | 14.5 [11.45-22.35] | 14.65 [10.97-18.99] | 0.67 |
| Salt (g) | 5.4 [3.9-7.19] | 4.825 [3.83-6.46] | 0.37 |
| Sodium (mg) | 2002 [1049.5-2792] | 1727 [1325.5-2337.38] | 0.72 |
| Potassium (mg) | 2695 [2072-3676.5] | 2433.5 [1936-3196.75] | 0.38 |
| Calcium (mg) | 650 [476.5-936.5] | 728.5 [606.75-941.75] | 0.24 |
| Magnesium (mg) | 236 [195.5-282.5] | 232 [189.25-296.25] | 0.80 |
| Iron (mg) | 9.3 [5.35-11.85] | 7.35 [5.61-10.12] | 0.41 |
| Selenium (µg) | 58 [37.5-75] | 49.5 [33.75-69.75] | 0.57 |
| Zinc (mg) | 8.45 [6.6-11.1] | 8.5 [7.02-10.6] | 0.89 |
| Vit A (µg) | 358 [222.5-578] | 435.5 [274.75-574.25] | 0.43 |
| Vit D (µg) | 2.6 [1.25-4.7] | 2.65 [1.6-4.93] | 0.85 |
| Vit E (mg) | 9.1 [5.7-14.82] | 8.05 [6.25-11.65] | 0.59 |
| Vit B1 (mg) | 1.04 [0.67-1.36] | 1 [0.7-1.25] | 0.84 |
| Vit B2 (mg) | 1.17 [0.81-1.84] | 1.38 [1.1-1.73] | 0.38 |
| Vit B6 (mg) | 1.67 [1.25-2.34] | 1.515 [1.09-1.78] | 0.12 |
| Folic acid (µg) | 154 [120-210] | 145 [106.75-192.5] | 0.80 |
| Vit B12 (µg) | 3.73 [2.05-6.8] | 4.32 [2.94-5.86] | 0.43 |
| Niacin (mg) | 20.7 [15.25-32.35] | 15.35 [11.08-27.05] | 0.12 |
| Vit C (mg) | 72 [38.5-98] | 50 [35.75-83.75] | 0.51 |
| Iodine (µg) | 117 [75-159.5] | 117.5 [96.5-153.5] | 0.64 |
| Phosphorus (mg) | 1341 [998-1665] | 1399.5 [1045.25-1666.75] | 0.76 |
| Sugar (g) | 76.4 [57-105.22] | 76.45 [63.05-96.25] | 0.95 |

IQR: interquartile range; *P values were determined through Mann-Whitney U tests for numerical variables and Fisher’s exact tests for categorical variables.*

*^$^ Sample size is indicated when missing data are present.*

*^#^* *Participants were asked to complete a 24-hour dietary recall diary to estimate food intake one day before the baseline study visits as previously described (15). Nutrient calculations were performed using the ‘eetmeter’ (i.e. ‘eating meter’ in English, developed by the ’Voedingscentrum’ of ‘Rijksinstituut voor Volksgezondheid en Milieu’ (RIVM) (16), to calculate the amount of energy consumption (in kcal), and fibers, fat, sugars, proteins, minerals, and vitamins (in grams). The mean nutrient calculations per day were calculated for subjects who filled in the intake for more than one day before inclusion. In total, dietary data were available for 85 children in SysPharmPediA, and 78 of the children included in this study had this data available (17).*

Table S4. Result of Mann-Whitney U test on influence of sex, OCS and biologics use on taxa and inflammatory mediators

|  | **Taxa** | **P-value** | **Inflammatory mediator** | **P-value** |
| --- | --- | --- | --- | --- |
| **Sex** | *Cardiobacterium* | 0.23 | VEGF | 0.74 |
|  | *Corynebacterium* | 0.31 | MIP-3β | 0.59 |
|  | *Capnocytophaga* | 0.30 | TIMP-4 | 0.64 |
| **Biologics** | *Cardiobacterium* | 0.60 | VEGF | 0.72 |
|  | *Corynebacterium* | 0.49 | MIP-3β | 0.50 |
|  | *Capnocytophaga* | 0.13 | TIMP-4 | 0.48 |
| **OCS** | *Cardiobacterium* | 0.91 | VEGF | 0.14 |
|  | *Corynebacterium* | 0.95 | MIP-3β | 0.97 |
|  | *Capnocytophaga* | 0.47 | TIMP-4 | 0.86 |

References

1. Andrews S. FastQC: A Quality Control Tool for High Throughput Sequence Data. 2010 [cited 2022 January 10th]. Available from: <http://www.bioinformatics.babraham.ac.uk/projects/fastqc/>.

2. Ewels P, Magnusson M, Lundin S, Kaller M. MultiQC: summarize analysis results for multiple tools and samples in a single report. *Bioinformatics* 2016; 32: 3047-3048.

3. Martin M. Cutadapt removes adapter sequences from high-throughput sequencing reads. *EMBnet journal* 2011; 17: 10-12.

4. Callahan BJ, McMurdie PJ, Rosen MJ, Han AW, Johnson AJ, Holmes SP. DADA2: High-resolution sample inference from Illumina amplicon data. *Nat Methods* 2016; 13: 581-583.

5. Quast C, Pruesse E, Yilmaz P, Gerken J, Schweer T, Yarza P, Peplies J, Glockner FO. The SILVA ribosomal RNA gene database project: improved data processing and web-based tools. *Nucleic Acids Res* 2013; 41: D590-596.

6. Mortensen MS, Brejnrod AD, Roggenbuck M, Abu Al-Soud W, Balle C, Krogfelt KA, Stokholm J, Thorsen J, Waage J, Rasmussen MA. The developing hypopharyngeal microbiota in early life. *Microbiome* 2016; 4: 1-12.

7. Wang Q, Garrity GM, Tiedje JM, Cole JR. Naive Bayesian classifier for rapid assignment of rRNA sequences into the new bacterial taxonomy. *Applied and environmental microbiology* 2007; 73: 5261-5267.

8. Davis NM, Proctor DM, Holmes SP, Relman DA, Callahan BJ. Simple statistical identification and removal of contaminant sequences in marker-gene and metagenomics data. *Microbiome* 2018; 6: 1-14.

9. Paulson JN, Stine OC, Bravo HC, Pop M. Differential abundance analysis for microbial marker-gene surveys. *Nature methods* 2013; 10: 1200-1202.

10. R Core Team. R: A language and environment for statistical computing. 2013.

11. McMurdie PJ, Holmes S. phyloseq: an R package for reproducible interactive analysis and graphics of microbiome census data. *PLoS One* 2013; 8: e61217.

12. Oksanen J, Blanchet FG, Kindt R, Legendre P, Minchin PR, O’hara R, Simpson GL, Solymos P, Stevens MHH, Wagner H. Package ‘vegan’. *Community ecology package, version* 2013; 2: 1-295.

13. Lin H, Peddada SD. Analysis of compositions of microbiomes with bias correction. *Nat Commun* 2020; 11: 3514.

14. Sharma D, Paterson AD, Xu W. TaxoNN: ensemble of neural networks on stratified microbiome data for disease prediction. *Bioinformatics* 2020; 36: 4544-4550.

15. Verster JC, Vermeulen SA, van de Loo AJ, Balikji S, Kraneveld AD, Garssen J, Scholey A. Dietary nutrient intake, alcohol metabolism, and hangover severity. *Journal of clinical medicine* 2019; 8: 1316.

16. Eetmeter. Available from: <https://mijn.voedingscentrum.nl/nl/eetmeter/>.

17. Abdel-Aziz MI, Hashimoto S, Neerincx AH, Haarman EG, Cecil A, Lintelmann J, Witting M, Hauck SM, Kerssemakers N, Verster JC. Metabotypes are linked to uncontrolled childhood asthma, gut microbiota, and systemic inflammation. *Journal of Allergy and Clinical Immunology* 2025.
