# Supplementary material for: Complementary Predictors for Asthma Attack Prediction in Children: Salivary Microbiome, Serum Inflammatory Mediators, and Past Attack History
Source: Allergy. 2025 Aug 18;81(2):413–26. doi: 10.1111/all.70004 (PMC12862560; doi:10.1111/all.70004)
Supplement: Supplementary file 3 — Appendix S3: all70004‐sup‐0003‐AppendixS3.pdf. [file ALL-81-413-s003.pdf]

U-BIOPRED

The U-BIOPRED consortium wishes to acknowledge the help and expertise of the following individuals and groups without whom, the study would not have been possible:

Definition for the U-BIOPRED Study Group Supplementary authors
Clinical site research leads
Platform leads
Data cleaning team
Data analysis team
Scientific Board and Management Board members
Core project management staff

Definition for the U-BIOPRED Study Group Contributors list
Significant involvement in the clinical study

Use of list:
This list is to be used for all non-core clinical papers.

Instructions
Follow up clinical papers should re-use the baseline cohort description paper lists, in order to recognize the clinical staff involved in the study.

**U-BIOPRED Supplementary authors**	
**Name**	**Affiliation**
Abdel-Aziz MI	Department of Respiratory Medicine, Amsterdam UMC, University of Amsterdam, Amsterdam, The Netherlands
Adcock I M	National Heart and Lung Institute, Imperial College, London, UK;
Andersson LI	Department of Respiratory Medicine, Karolinska University Hospital, Stockholm, Sweden
Auffray C	European Institute for Systems Biology and Medicine, CNRS-ENS-UCBL-INSERM, Lyon, France;
Badi YE,	National Heart and Lung Institute, Imperial College, London, UK;
Bakke P	Department of Clinical Science, University of Bergen, Bergen, Norway;
Bansal A T	Acclarogen Ltd, St. John's Innovation Centre, Cambridge, UK;
Baribaud F	Janssen R&D, LLC, Spring House, PA, USA
Bates S	Respiratory Therapeutic Unit, GSK, London, UK;
Bel E H	Academic Medical Centre, University of Amsterdam, Amsterdam, The Netherlands;
Bigler J	*Previously Amgen Inc*
Billing B	Department of Respiratory Medicine, Karolinska University Hospital, Stockholm, Sweden
Bisgaard H	COPSAC, Copenhagen Prospective Studies on Asthma in Childhood, Herlev and Gentofte Hospital, University of Copenhagen, Copenhagen, Denmark
Boedigheimer M J	Amgen Inc.; Thousand Oaks, USA
Bønnelykke K	COPSAC, Copenhagen Prospective Studies on Asthma in Childhood, Herlev and Gentofte Hospital, University of Copenhagen, Copenhagen, Denmark;
Brandsma J	University of Southampton, Southampton, UK
Brinkman P	Academic Medical Centre, University of Amsterdam, Amsterdam, The Netherlands;
Bucchioni E	Chiesi Pharmaceuticals SPA, Parma, Italy
Burg D	Centre for Proteomic Research, Institute for Life Sciences, University of Southampton, Southampton, UK

U-BIOPRED

Bush A	National Heart and Lung Institute, Imperial College, London, UK; Royal Brompton and Harefield NHS trust, UK
Caruso M	Dept. Biomedical and Biotechnological Sciences, University of Catania, Catania, Italy;
Chalekis R	Institute of Environmental Medicine, Centre for Allergy Research, Karolinska Institutet, Stockholm, Sweden
Chanez P	Assistance publique des Hôpitaux de Marseille - Clinique des bronches, allergies et sommeil, Aix Marseille Université, Marseille, France
Chung F K	National Heart and Lung Institute, Imperial College, London, UK;
Checa T	Institute of Environmental Medicine, Centre for Allergy Research, Karolinska Institutet, Stockholm, Sweden
Compton C H	Respiratory Therapeutic Unit, GSK, London, UK
Corfield J	Areteva R&D, Nottingham, UK;
Cunoosamy D	Sanofi, Cambridge, USA
D'Amico A	University of Rome 'Tor Vergata', Rome Italy;
Dahlén B	Department of Respiratory Medicine, Karolinska University Hospital & Centre for Allergy Research, Karolinska Institutet, Stockholm, Sweden
Dahlén S E	Institute of Environmental Medicine, Centre for Allergy Research, Karolinska Institutet, and Department of Respiratory Medicine, Karolinska University Hospital, Stockholm, Sweden
De Meulder B	European Institute for Systems Biology and Medicine, CNRS-ENS-UCBL-INSERM, Lyon, France;
Djukanovic R	NIHR Southampton Respiratory Biomedical Research Unit and Clinical and Experimental Sciences, Southampton, UK;
Erpenbeck V J	Translational Medicine, Respiratory Profiling, Novartis Institutes for Biomedical Research, Basel, Switzerland;
Erzen D	Boehringer Ingelheim Pharma GmbH & Co. KG; Biberach, Germany
Fichtner K	Boehringer Ingelheim Pharma GmbH & Co. KG; Biberach, Germany
Fleming L J	National Heart and Lung Institute, Imperial College, London, UK; Royal Brompton and Harefield NHS trust, UK
Formaggio E	*Previously CROMSOURCE, Verona Italy*
Fowler S J	Division of infection, immunity and respiratory medicine, School of biological sciences, University of Manchester, Manchester University NHS Foundation Trust, Manchester Academic Health Science Centre, Manchester, United Kingdom
Frey U	University Children's Hospital, Basel, Switzerland;
Gahlemann M	Boehringer Ingelheim (Schweiz) GmbH,Basel, Switzerland;
Geiser T	Department of Respiratory Medicine, University Hospital Bern, Switzerland;
Goss V	NIHR Respiratory Biomedical Research Unit, University Hospital Southampton NHS Foundation Trust, Integrative Physiology and Critical Illness Group, Clinical and Experimental Sciences, Sir Henry Wellcome Laboratories, Faculty of Medicine, University of Southampton, Southampton, UK;
Guo Y	Data Science Institute, Imperial College, London, UK;
Hashimoto S	Academic Medical Centre, University of Amsterdam, Amsterdam, The Netherlands;
Haughney J	International Primary Care Respiratory Group, Aberdeen, Scotland;
Hedlin G	Dept. Women's and Children's Health & Centre for Allergy Research, Karolinska Institutet, Stockholm, Sweden;
Hekking P W	Academic Medical Centre, University of Amsterdam, Amsterdam, The Netherlands;
Higenbottam T	Allergy Therapeutics, West Sussex, UK;

Hohlfeld J M	Fraunhofer Institute for Toxicology and Experimental Medicine, Hannover, Germany
Holweg C	Respiratory and Allergy Diseases, Genentech, San Francisco, USA
Horváth I	Department of Pulmonology and Department of Public Health, Semmelweis University, Budapest, Hungary.
Howarth P	NIHR Southampton Respiratory Biomedical Research Unit, Clinical and Experimental Sciences and Human Development and Health, Southampton, UK
James A J	Institute of Environmental Medicine, Centre for Allergy Research, Karolinska Institutet, Stockholm, Sweden;
Knowles RG	Knowles Consulting Ltd, Stevenage. UK;
Kolmert J	Institute of Environmental Medicine, Centre for Allergy Research, Karolinska Institutet, Stockholm, Sweden
Konradsen J	Dept. Women's and Children's Health & Centre for Allergy Research, Karolinska Institutet, Stockholm, Sweden
Krug N	Fraunhofer Institute for Toxicology and Experimental Medicine, Hannover, Germany;
Lazarinis N	Department of Respiratory Medicine, Karolinska University Hospital & Centre for Allergy Research, Karolinska Institutet, Stockholm, Sweden
Li C-X	Department of Medicine Solna, Karolinska Institutet, Stockholm, Sweden
Loza M J	Janssen R&D, LLC, Spring House, PA, USA
Lutter R	Academic Medical Centre, University of Amsterdam, Amsterdam, The Netherlands;
Manta A	Roche Diagnostics GmbH, Mannheim, Germany
Masefield S	European Lung Foundation, Sheffield, UK;
Maitland-van der Zee Anke-Hilse	Department of Respiratory Medicine, Amsterdam UMC, University of Amsterdam, Amsterdam, The Netherlands;
Matthews J G	Respiratory and Allergy Diseases, Genentech, San Francisco, USA;
Mazein A	European Institute for Systems Biology and Medicine, CNRS-ENS-UCBL-INSERM, Lyon, France
Middelveld R J M	Centre for Allergy Research, Karolinska Institutet, Stockholm, Sweden
Miralpeix M	Almirall, Barcelona, Spain;
Montuschi P	Università Cattolica del Sacro Cuore, Milan, Italy;
Murray C S	Division of infection, immunity and respiratory medicine, School of biological sciences, University of Manchester, Manchester University NHS Foundation Trust, and Manchester Academic Health Science Centre, Manchester, United Kingdom
Musial J	Dept. of Medicine, Jagiellonian University Medical College, Krakow, Poland
Mumby, S	National Heart and Lung Institute, Imperial College, London, UK
Myles D	Respiratory Therapeutic Unit, GSK, London, UK;
Nordlund B	Dept. Women's and Children's Health & Centre for Allergy Research, Karolinska Institutet, Stockholm, Sweden
Pandis I	Data Science Institute, Imperial College, London, UK
Pavlidis S	National Heart and Lung Institute, Imperial College, London, UK
Postle A	University of Southampton, UK
Powel P	European Lung Foundation, Sheffield, UK;
Praticò G	CROMSOURCE, Verona, Italy
Puig Valls M	CROMSOURCE, Barcelona, Spain
Rao N	Janssen R&D, LLC, Spring House, PA, USA
Reinke S	Institute of Environmental Medicine, Karolinska Institutet, Stockoholm, Sweden
Riley J	Respiratory Therapeutic Unit, GSK, London, UK;
Roberts A	Asthma UK, London, UK;

Roberts G	NIHR Southampton Respiratory Biomedical Research Unit, Clinical and Experimental Sciences and Human Development and Health, Southampton, UK;
Rowe A	Janssen R&D, UK;
Sandström T	Dept of Public Health and Clinical Medicine, Umeå University, Umeå, Sweden;
Schofield JPR	Centre for Proteomic Research, Institute for Life Sciences, University of Southampton, Southampton, UK
Seibold W	Boehringer Ingelheim Pharma GmbH, Biberach, Germany
Shaw D E	Respiratory Research Unit, University of Nottingham, UK;
Sigmund R	Boehringer Ingelheim Pharma GmbH & Co. KG; Biberach, Germany
Singer F	Pediatric Respiratory Medicine, Department of Pediatrics, Inselspital, Bern University Hospital, University of Bern, Bern, Switzerland.
Skipp P J	Centre for Proteomic Research, Institute for Life Sciences, University of Southampton, Southampton, UK
Smicker M	Sanofi, Cambridge, USA
Sousa A R	Respiratory Therapeutic Unit, GSK, London, UK;
Sparreman-Mikus M	Department of Medicine Huddinge, Karolinska Institutet, and Department of Respiratory Medicine, Karolinska University Hospital, Stockholm, Sweden
Sterk P J	Academic Medical Centre, University of Amsterdam, Amsterdam, The Netherlands;
Ström M	Department of Medicine Solna, Karolinska Institutet, Stockholm, Sweden
Sun K	Data Science Institute, Imperial College, London, UK
Thornton B	MSD, USA
Uddin M	AstraZeneca BioPharmaceuticals R&D, Gothenburg, Sweden
Versi A	National Heart and Lung Institute, Imperial College, London, UK
Vestbo J	Centre for Respiratory Medicine and Allergy, Institute of Inflammation and Repair, University of Manchester and University Hospital of South Manchester, Manchester Academic Health Sciences Centre, Manchester, United Kingdom
Vissing N H	COPSAC, Copenhagen Prospective Studies on Asthma in Childhood, Herlev and Gentofte Hospital, University of Copenhagen, Copenhagen, Denmark;
Wagers S S	BioSci Consulting, Maasmechelen, Belgium
Wheelock A M	Respiratory Medicine Unit, Department of Medicine Solna and Center for Molecular Medicine, Karolinska Institutet, Stockholm, Sweden; and Department of Respiratory Medicine and Allergy, Karolinska University Hospital Solna, Stockholm, Sweden;
Wheelock C E	Institute of Environmental Medicine, Centre for Allergy Research, Karolinska Institutet, Stockholm, Sweden;
Wilson S J	Histochemistry Research Unit, Faculty of Medicine, University of Southampton, Southampton, UK;
Yasinska V	Department of Medicine Huddinge, Karolinska Institutet, and Department of Respiratory Medicine, Karolinska University Hospital, Stockholm, Sweden
Zounemat Kermani, N	Data Science Institute, Imperial College, London, UK

Contributors

Ahmed H, European Institute for Systems Biology and Medicine, CNRS-ENS-UCBL-INSERM, Lyon, France;
Aliprantis Antonios, Merck Research Laboratories, Boston, USA;

Allen David, North West Severe Asthma Network, Pennine Acute Hospital NHS Trust, UK
Alving Kjell, Dept Women's & Children's Health, Uppsala University, Uppsala, Sweden
Badorrek P, Fraunhofer ITEM; Hannover, Germany
Balgoma David, Centre for Allergy Research, Karolinska Institutet, Stockholm, Sweden
Ballereau S, European institute for Systems Biology and Medicine, University of Lyon, France
Barber Clair, NIHR Southampton Respiratory Biomedical Research Unit and Clinical and Experimental Sciences, Southampton, UK;
Batuwitage Manohara Kanangana, Data Science Institute, Imperial College, London, UK
Bautmans An, MSD, Brussels, Belgium
Bedding A, Roche Diagnostics GmbH, Mannheim, Germany
Behndig AF, Umeå University, Umea, Sweden
Beleta Jorge, Almirall S.A., Barcelona, Spain;
Berglind A, MSD, Brussels, Belgium
Bochenek Grazyna, II Department of Internal Medicine, Jagiellonian University Medical College, Krakow, Poland;
Braun Armin, Fraunhofer Institute for Toxicology and Experimental Medicine, Hannover, Germany;
Campagna D, Department of Clinical and Experimental Medicine, University of Catania, Catania, Italy;
Carayannopoulos Leon, Previously at: MSD, USA;
Casaulta C, University Children's Hospital of Bern, Switzerland
Chaiboonchoe A, European Institute for Systems Biology and Medicine, CNRS-ENS-UCBL-INSERM, Lyon, France;
Chaleckis Romanas, Centre of Allergy Research, Karolinska Institutet, Stockholm, Sweden
Davison Timothy Janssen R&D, LLC, Spring House, PA, USA
De Alba Jorge, Almirall S.A., Barcelona, Spain;
De Lepeleire Inge, MSD, Brussels, BE
Dekker Tamara, Academic Medical Centre, University of Amsterdam, Amsterdam, The Netherlands;
Delin Ingrid, Centre for Allergy Research, Karolinska Institutet, Stockholm, Sweden
Dennison P, NIHR Southampton Respiratory Biomedical Research Unit, Clinical and Experimental Sciences, NIHR-Wellcome Trust Clinical Research Facility, Faculty of Medicine, University of Southampton, Southampton, UK;
Dijkhuis Annemiek, Academic Medical Centre, University of Amsterdam, Amsterdam, The Netherlands;
Dodson Paul, AstraZeneca BioPharmaceuticals R&D, Gothenburg, Sweden
Draper Aleksandra, BioSci Consulting, Maasmechelen, Belgium;
Dyson K, CROMSOURCE; Stirling, UK
Edwards Jessica, Asthma UK, London, UK;
El Hadjam L, European Institute for Systems Biology and Medicine, University of Lyon
Emma Rosalia, Department of Biomedical and Biotechnological Sciences, University of Catania, Catania, Italy;
Ericsson Magnus, Karolinska University Hospital, Stockholm, Sweden

Faulenbach C, Fraunhofer ITEM; Hannover, Germany
Flood Breda, European Federation of Allergy and Airways Diseases Patient's Associations, Brussels, Belgium
Galffy G, Semmelweis University, Budapest, Hungary;
Gallart Hector, Centre for Allergy Research, Karolinska Institutet, Stockholm, Sweden
Garissi D, Global Head Clinical Research Division, CROMSOURCE, Italy
Gent J, Royal Brompton and Harefield NHS Foundation Trust, London, UK;
Gerhardsson de Verdier M, AstraZeneca BioPharmaceuticals R&D, Gothenburg, Sweden
Gibeon D, National Heart and Lung Institute, Imperial College, London, UK;
Gomez Cristina, Centre for Allergy Research, Karolinska Institutet, Stockholm, Sweden
Gove Kerry, NIHR Southampton Respiratory Biomedical Research Unit and Clinical and Experimental Sciences, Southampton, UK;
Gozzard Neil, UCB, Slough, UK;
Guillmant-Farry E, Royal Brompton Hospital, London, UK
Henriksson E, Karolinska University Hospital & Karolinska Institutet, Stockholm, Sweden
Hewitt Lorraine, NIHR Southampton Respiratory Biomedical Research Unit, Southampton, UK
Hoda U, Imperial College, London, UK
Hu Richard, Amgen Inc. Thousand Oaks, USA
Hu Sile, National Heart and Lung Institute, Imperial College, London, UK;
Hu X, Amgen Inc.; Thousand Oaks, USA
Jeyasingham E, UK Clinical Operations, GSK, Stockley Park, UK
Johnson K, Centre for respiratory medicine and allergy, Institute of Inflammation and repair, University Hospital of South Manchester, NHS Foundation Trust, Manchester, UK
Jullian N, European Institute for Systems Biology and Medicine, University of Lyon
Kamphuis Juliette, Longfonds, Amersfoort, The Netherlands;
Kennington Erika J., Asthma UK, London, UK;
Kerry Dyson, CromSource, Stirling, UK;
Kerry G, Centre for respiratory medicine and allergy, Institute of Inflammation and repair, University Hospital of South Manchester, NHS Foundation Trust, Manchester, UK
Klüglich M, Boehringer Ingelheim Pharma GmbH & Co. KG; Biberach, Germany
Knobel Hugo, Philips Research Laboratories, Eindhoven, The Netherlands;
Knox Alan J, Respiratory Research Unit, University of Nottingham, Nottingham, UK;
Kolmert Johan, Centre for Allergy Research, Karolinska Institutet, Stockholm, Sweden
Konradsen J R, Dept. Women's and Children's Health & Centre for Allergy Research, Karolinska Institutet, Stockholm, Sweden
Kots Maxim, Chiesi Pharmaceuticals, SPA, Parma, Italy;
Kretsos Kosmas, UCB, Slough, UK
Krueger L, University Children's Hospital Bern, Switzerland
Kuo Scott, National Heart and Lung Institute, Imperial College, London, UK;
Kupczyk Maciej, Centre for Allergy Research, Karolinska Institutet, Stockholm, Sweden
Lambrecht Bart, University of Gent, Gent, Belgium;

Lantz A-S, Karolinska University Hospital & Centre for Allergy Research, Karolinska Institutet, Stockholm, Sweden
Larminie Christopher, GSK, London, UK
Larsson L X, AstraZeneca BioPharmaceuticals R&D, Gothenburg, Sweden
Latzin P, University Children’s Hospital of Bern, Bern, Switzerland
Lazarinis N, Karolinska University Hospital & Karolinska Institutet, Stockholm, Sweden
Lefaudeux Diane, European Institute for Systems Biology and Medicine, CNRS-ENS-UCBL-INSERM, Lyon, France;
Lemonnier N, European Institute for Systems Biology and Medicine, CNRS-ENS-UCBL-INSERM, Lyon, France
Li Chuan-Xing, Respiratory Medicine Unit, Department of Medicine Solna and Center for Molecular Medicine, Karolinska Institutet, Stockholm, Sweden; and Department of Respiratory Medicine and Allergy, Karolinska University Hospital Solna, Stockholm, Sweden;
Lone-Latif Saeeda, Academic Medical Centre, University of Amsterdam, Amsterdam, The Netherlands;
Lowe L A, Centre for respiratory medicine and allergy, Institute of Inflammation and repair, University Hospital of South Manchester, NHS Foundation Trust, Manchester, UK
Manta Alexander, Roche Diagnostics GmbH, Mannheim, Germany
Marouzet Lisa, NIHR Southampton Respiratory Biomedical Research Unit, Southampton, UK
Martin Jane, NIHR Southampton Respiratory Biomedical Research Unit, Southampton, UK
Mathon Caroline, Centre of Allergy Research, Karolinska Institutet, Stockholm, Sweden
McEvoy L, University Hospital, Department of Pulmonary Medicine, Bern, Switzerland
Meah Sally, National Heart and Lung Institute, Imperial College, London, UK;
Menzies-Gow A, Royal Brompton and Harefield NHS Foundation Trust, London, UK;
Metcalf Leanne, Previously at: Asthma UK, London, UK;
Meiser Andrea, Data Science Institute, Imperial College, London, UK
Mikus Maria, Science for Life Laboratory & The Royal Institute of Technology, Stockholm, Sweden;
Monk Philip, Synairgen Research Ltd, Southampton, UK;
Mores N, Università Cattolica del Sacro Cuore, Milan, Italy;
Naz Shama, Centre for Allergy Research, Karolinska Institutet, Stockholm, Sweden
Nething K, Boehringer Ingelheim Pharma GmbH & Co. KG; Biberach, Germany
Nicholas Ben, University of Southampton, Southampton, UK
Nihlén U, *Previously* AstraZeneca BioPharmaceuticals R&D, Gothenburg, Sweden
Nilsson Peter, Science for Life Laboratory & The Royal Institute of Technology, Stockholm, Sweden;
Niven R, North West Severe Asthma Network, University Hospital South Manchester, UK
Nordlund B, Dept. Women’s and Children’s Health & Centre for Allergy Research, Karolinska Institutet, Stockholm, Sweden
Nsubuga S, Royal Brompton Hospital, London, UK
Pacino Antonio, Lega Italiano Anti Fumo, Catania, Italy;
Palkonen Susanna, European Federation of Allergy and Airways Diseases Patient’s Associations, Brussels, Belgium.

Pahus L, Assistance publique des Hôpitaux de Marseille, Clinique des bronches, allergies et sommeil Espace Éthique Méditerranéen, Aix-Marseille Université, Marseille, France
Pellet J, European Institute for Systems Biology and Medicine, CNRS-ENS-UCBL-INSERM, Lyon, France
Pennazza Giorgio, Unit of Electronics for Sensor Systems, Department of Engineering, Campus Bio-Medico University of Rome, Rome, Italy
Petrén Anne, Centre for Allergy Research, Karolinska Institutet, Stockholm, Sweden
Pink Sandy, NIHR Southampton Respiratory Biomedical Research Unit, Southampton, UK
Pison C, European Institute for Systems Biology and Medicine, CNRS-ENS-UCBL-INSERM, Lyon, France
Rahman-Amin Malayka, Previously at: Asthma UK, London, UK;
Ravanetti Lara, Academic Medical Centre, University of Amsterdam, Amsterdam, The Netherlands;
Ray Emma, NIHR Southampton Respiratory Biomedical Research Unit, Southampton, UK
Reinke Stacey, Centre for Allergy Research, Karolinska Institutet, Stockholm, Sweden
Reynolds Leanne, Previously at: Asthma UK, London, UK;
Riemann K, Boehringer Ingelheim Pharma GmbH & Co. KG; Biberach, Germany
Robberechts Martine, MSD, Brussels, Belgium
Rocha J P, Royal Brompton and Harefield NHS Foundation Trust
Rossios C, National Heart and Lung Institute, Imperial College, London, UK;
Russell Kirsty, National Heart and Lung Institute, Imperial College, London, UK;
Rutgers Michael, Longfonds, Amersfoort, The Netherlands;
Santini G, Università Cattolica del Sacro Cuore, Milan, Italy;
Santonico Marco, Unit of Electronics for Sensor Systems, Department of Engineering, Campus Bio-Medico University of Rome, Rome, Italy
Saqi M, European Institute for Systems Biology and Medicine, CNRS-ENS-UCBL-INSERM, Lyon, France
Schoelch Corinna, Boehringer Ingelheim Pharma GmbH & Co. KG, Biberach, Germany
Scott S, North West Severe Asthma Network, Countess of Chester Hospital, UK
Sehgal N, North West Severe Asthma Network; Pennine Acute Hospital NHS Trust
Selby A, NIHR Southampton Respiratory Biomedical Research Unit, Clinical and Experimental Sciences and Human Development and Health, Southampton, UK;
Sjödin Marcus, Centre for Allergy Research, Karolinska Institutet, Stockholm, Sweden
Smids Barbara, Academic Medical Centre, University of Amsterdam, Amsterdam, The Netherlands;
Smith Caroline, NIHR Southampton Respiratory Biomedical Research Unit, Southampton, UK
Smith Jessica, Asthma UK, London, UK;
Smith Katherine M., University of Nottingham, UK;
Söderman P, Dept. Women's and Children's Health, Karolinska Institutet, Stockholm, Sweden
Sogbesan A, Royal Brompton and Harefield NHS Foundation Trust, London, UK;
Spycher F, University Hospital Department of Pulmonary Medicine, Bern, Switzerland
Staykova Doroteya, University of Southampton, Southampton, UK

U-BIOPRED

Stephan S, Centre for respiratory medicine and allergy, Institute of Inflammation and repair, University Hospital of South Manchester, NHS Foundation Trust, Manchester, UK
Stokholm J, University of Copenhagen and Danish Pediatric Asthma Centre Denmark
Strandberg K, Karolinska University Hospital & Karolinska Institutet, Stockholm, Sweden
Sunther M, Centre for respiratory medicine and allergy, Institute of Inflammation and repair, University Hospital of South Manchester, NHS Foundation Trust, Manchester, UK
Szentkereszty M, Semmelweis University, Budapest, Hungary;
Tamasi L, Semmelweis University, Budapest, Hungary;
Tariq K, NIHR Southampton Respiratory Biomedical Research Unit, Clinical and Experimental Sciences, NIHR-Wellcome Trust Clinical Research Facility, Faculty of Medicine, University of Southampton, Southampton, UK;
Thörngren John-Olof, Karolinska University Hospital, Stockholm, Sweden
Thorsen Jonathan, COPSAC, Copenhagen Prospective Studies on Asthma in Childhood, Herlev and Gentofte<br>Hospital, University of Copenhagen, Copenhagen, Denmark;
Valente S, Università Cattolica del Sacro Cuore, Milan, Italy;
van Aalderen W M, Academic Medical Centre, University of Amsterdam, Amsterdam, The Netherlands
van de Pol Marianne, Academic Medical Centre, University of Amsterdam, Amsterdam ,The Netherlands;
van Drunen C M, Academic Medical Centre, University of Amsterdam, Amsterdam, The Netherlands;
Van Eyll Jonathan, UCB, Slough, UK
Versnel Jenny, Previously at: Asthma UK, London, UK;
Vink Anton, Philips Research Laboratories, Eindhoven, The Netherlands;
von Garnier C, University Hospital Bern, Switzerland;
Vyas A, North west Severe Asthma Network, Lancashire Teaching Hospitals NHS Trust, UK
Wagener A H, Academic Medical Center Amsterdam, Amsterdam, The Netherlands
Wald Frans, Boehringer Ingelheim Pharma GmbH & Co. KG, Biberach, Germany
Walker Samantha, Asthma UK, London, UK;
Ward Jonathan, Histochemistry Research Unit, Faculty of Medicine, University of Southampton, Southampton, UK;
Wetzel Kristiane, Boehringer Ingelheim Pharma GmbH, Biberach, Germany
Wiegman Coen, National Heart and Lung Institute, Imperial College, London, UK;
Weiszhart Z, Semmelweis University, Budapest, Hungary
Williams Siân, International Primary Care Respiratory Group, Aberdeen, Scotland;
Yang Xian, Data Science Institute, Imperial College, London, UK
Yeyasingham Elizabeth, UK Clinical Operations, GSK, Stockley Park, UK;
Yu W, Amgen Inc.; Thousand Oaks, USA
Zetterquist W, Dept. Women's and Children's Health & Centre for Allergy Research, Karolinska Institutet, Stockholm, Sweden
Zolkipli Z, NIHR Southampton Respiratory Biomedical Research Unit, Clinical and Experimental Sciences and Human Development and Health, Southampton, UK;

Zwinderman A H, Academic Medical Centre, University of Amsterdam, The Netherlands;

Partner organisations	
Novartis Pharma AG	University of Southampton, Southampton, UK
Academic Medical Centre, University of Amsterdam, Amsterdam, The Netherlands	Imperial College London, London, UK
University of Catania, Catania, Italy	University of Rome 'Tor Vergata', Rome, Italy
Hvidore Hospital, Hvidore, Denmark	Jagiellonian Univ. Medi.College, Krakow, Poland
University Hospital, Inselspital, Bern, Switzerland	Semmelweis University, Budapest, Hungary
University of Manchester, Manchester, UK	Université d'Aix-Marseille, Marseille, France
Fraunhofer Institute, Hannover, Germany	University Hospital, Umea, Sweden
Ghent University, Ghent, Belgium	Ctr. Nat. Recherche Scientifique, Lyon, France
Università Cattolica del Sacro Cuore, Rome, Italy	University Hospital, Copenhagen, Denmark
Karolinska Institutet, Stockholm, Sweden	Nottingham University Hospital, Nottingham, UK
University of Bergen, Bergen, Norway	Netherlands Asthma Foundation, Leusden, NL
European Lung Foundation, Sheffield, UK	Asthma UK, London, UK
European. Fed. of Allergy and Airways Diseases Patients' Associations, Brussels, Belgium	Lega Italiano Anti Fumo, Catania, Italy
International Primary Care Respiratory Group, Aberdeen, Scotland	Philips Research Laboratories, Eindhoven, NL
Synairgen Research Ltd, Southampton, UK	Aerocrine AB, Stockholm, Sweden
BioSci Consulting, Maasmechelen, Belgium	Almirall
AstraZeneca BioPharmaceuticals R&D	Boehringer Ingelheim
Chiesi	GlaxoSmithKline
Roche	UCB
Janssen Biologics BV	Amgen NV
Merck Sharp & Dome Corp	

MEMBERS OF THE ETHICS BOARD			
**Name**	**Task**	**Affiliation**	**e-mail**
Jan-Bas Prins	Biomedical research	LUMC/the Netherlands	J.B.Prins@lumc.nl
Martina Gahlemann	Clinical care	BI/Germany	Martina.Gahlemann@boehringer-ingelheim.com
Luigi Visintin	Legal affairs	LIAF/Italy	visintin@inrete.it
Hazel Evans	Paediatric care	Southampton/UK	hazel.evans@uhs.nhs.uk
Martine Puhl	Patient representation (co-chair)	NAF/ the Netherlands	martine@puhl.nl
Lina Buzermaniene	Patient representation	EFA/Lithuania	lina.buzermaniene@pavb.lt
Val Hudson	Patient representation	Asthma UK	hudsonval7@gmail.com
Laura Bond	Patient representation	Asthma UK	lvbond22@googlemail.com
Pim de Boer	Patient representation and pathobiology	IND	deboer.pim@hetnet.nl

Guy Widdershoven	Research ethics	VUMC/the Netherlands	g.widdershoven@vumc.nl
Ralf Sigmund	Research methodology and biostatistics	BI/Germany	ralf.sigmund@boehringer-ingelheim.com

THE PATIENT INPUT PLATFORM	
**Name**	**Country**
Amanda Roberts	UK
David Supple (chair)	UK
Dominique Hamerlijnck	The Netherlands
Jenny Negus	UK
Juliëtte Kamphuis	The Netherlands
Lehanne Sergison	UK
Luigi Visintin	Italy
Pim de Boer (co-chair)	The Netherlands
Susanne Onstein	The Netherlands

MEMBERS OF THE SAFETY MONITORING BOARD	
**Name**	**Task**
William MacNee	Clinical care
Renato Bernardini	Clinical pharmacology
Louis Bont	Paediatric care and infectious diseases
Per-Ake Wecksell	Patient representation
Pim de Boer	Patient representation and pathobiology (chair)
Martina Gahlemann	Patient safety advice and clinical care (co-chair)
Ralf Sigmund	Bio-informatician
